# Drug-Induced Photosensitivity—From Light and Chemistry to Biological Reactions and Clinical Symptoms

**DOI:** 10.3390/ph14080723

**Published:** 2021-07-26

**Authors:** Justyna Kowalska, Jakub Rok, Zuzanna Rzepka, Dorota Wrześniok

**Affiliations:** Department of Pharmaceutical Chemistry, Faculty of Pharmaceutical Sciences in Sosnowiec, Medical University of Silesia in Katowice, Jagiellońska 4, 41-200 Sosnowiec, Poland; jkowalska@sum.edu.pl (J.K.); jrok@sum.edu.pl (J.R.); zrzepka@sum.edu.pl (Z.R.)

**Keywords:** phototoxicity, photoallergy, melanin, oxidative stress, photodegradation

## Abstract

Photosensitivity is one of the most common cutaneous adverse drug reactions. There are two types of drug-induced photosensitivity: photoallergy and phototoxicity. Currently, the number of photosensitization cases is constantly increasing due to excessive exposure to sunlight, the aesthetic value of a tan, and the increasing number of photosensitizing substances in food, dietary supplements, and pharmaceutical and cosmetic products. The risk of photosensitivity reactions relates to several hundred externally and systemically administered drugs, including nonsteroidal anti-inflammatory, cardiovascular, psychotropic, antimicrobial, antihyperlipidemic, and antineoplastic drugs. Photosensitivity reactions often lead to hospitalization, additional treatment, medical management, decrease in patient’s comfort, and the limitations of drug usage. Mechanisms of drug-induced photosensitivity are complex and are observed at a cellular, molecular, and biochemical level. Photoexcitation and photoconversion of drugs trigger multidirectional biological reactions, including oxidative stress, inflammation, and changes in melanin synthesis. These effects contribute to the appearance of the following symptoms: erythema, swelling, blisters, exudation, peeling, burning, itching, and hyperpigmentation of the skin. This article reviews in detail the chemical and biological basis of drug-induced photosensitivity. The following factors are considered: the chemical properties, the influence of individual ranges of sunlight, the presence of melanin biopolymers, and the defense mechanisms of particular types of tested cells.

## 1. Introduction: Photosensitivity as an Adverse Drug Reaction

The World Health Organization defines an adverse drug reaction (ADR) as “a response to a drug which is noxious and unintended, and which occurs at doses normally used in man for the prophylaxis, diagnosis, or therapy of disease, or the modifications of physiological function”. The majority of ADRs (about 75–80%) are predictable, nonimmunologic, usually dose-dependent, and related to the drug pharmacology [1,2]. Cutaneous adverse drug reactions are thought to be one of the most frequently occurring ADRs [3,4]. They often require hospitalization, additional treatment, medical management, and generate significant costs for the payer as well as for the service provider [5,6,7]. In addition to the above, they also lead to a decrease in patient’s comfort and the limitation of drug usage.

Photosensitivity belongs to the most common type of skin-related adverse drug reactions. Taking into account the causes and mechanisms, photoallergic and phototoxic reactions are distinguished among drug-induced photosensitization. Currently, the number of photosensitization cases is constantly increasing. The reasons for this can be found in excessive exposure to sunlight, dictated by the aesthetic value of a tan, and the increasing number of photosensitizing substances in food and dietary supplements as well as pharmaceutical and cosmetic products. Medicines constitute a large percentage of photosensitizing substances, including those available without a prescription (OTC). The risk of phototoxic and photoallergic reactions concerns several hundred currently used drugs, including antibiotics and other antimicrobial drugs, nonsteroidal anti-inflammatory drugs, drugs used in the pharmacotherapy of cardiovascular diseases (diuretics, antiarrhythmics, antihypertensive drugs), drugs acting on the central nervous system (neuroleptics, antidepressants), antidiabetic, and anticancer drugs [8,9]. Symptoms of drug-induced photosensitivity usually appear up to several hours after exposure to sunlight and include erythema, swelling, blisters, exudation, peeling, burning, itching, and hyperpigmentation of the skin. These changes are observed directly in places exposed to solar radiation [10].

## 2. Drug Photosafety Assessment

Studies of drug photosensitivity are an important part of the safety assessment of medicinal products. They are used to identify the potential risks that medications can cause in combination with exposure to sunlight. The assessment of photosafety should be required for new active pharmaceutical ingredients, as well as for new excipients, topical and transdermal pharmaceutical formulations, and photodynamic therapeutics.

The applied methods of drug phototoxicity tests are defined by the relevant guidelines of medicine and drug agencies, e.g., the Food and Drug Administration (FDA) [11] and the European Medicine Agency (EMA) [12]. These guidelines are based on ICH (The International Council for Harmonisation of Technical Requirements for Pharmaceuticals for Human Use) Guidance on Photosafety Evaluation of Pharmaceuticals [13]. Nowadays, 3T3 Neutral Red Uptake (NRU) test is the most recommended and appropriate in vitro method to assess phototoxic potential. The test uses an immortalized mouse fibroblast cell line called Balb/c 3T3. The method compares the cytotoxicity of a chemical on unirradiated cells and cells exposed to a noncytotoxic dose of UV-Vis radiation. Differences in the neutral red uptake can be measured with a spectrophotometer, which allows the distinction between viable, damaged, or dead cells.

The estimated specificity of the 3T3 NRU test is 84%. However, practical experience from the pharmaceutical industry suggests a much lower specificity. The 3T3 NRU test detects phototoxic properties of an evaluated substance, however, it has some serious limitations. Since the 3T3 cell line is sensitive to UVB, the recommended irradiation conditions involve the use of filters to attenuate wavelengths below 320 nm. Furthermore, the assay does not determine the cellular, molecular, and biochemical mechanisms and effects of the phototoxic action accurately.

## 3. Chemical and Biological Basis of Photosensitivity

The occurrence of drug-induced photosensitivity reactions depends both on the properties of the medicament and the exposure to UV-vis radiation. Photosensitizing drugs are radiation-absorbing compounds. Electromagnetic radiation emitted by the Sun includes three components: ultraviolet (UV) light (180–380 nm), visible light (380–700 nm), and infrared (IR) rays (700 nm–3 µm). It has been found that 6.8% of the sunlight is UV, 38.9% is visible light, and 54.3% is infrared (IR) [14,15]. Taking into account the problem of drug-induced photosensitivity, radiation in the visible and ultraviolet A and B ranges is of significant relevance. Visible wavelengths show deep skin penetrance and can influence structures in the epidermis, dermis, and subcutis [16]. UV light is divided into three regions: UVC (180–280 nm), UVB (280–320 nm), and UVA (320–380 nm). UVC, the most energetic part of UV light, does not affect the skin because it is absorbed by the ozone layer [14]. UV radiation reaching the skin’s surface comprises approximately 5% UVB and 95% UVA [17]. Shorter UVB wavelengths penetrate into the epidermis, whereas UVA radiation reaches the papillary dermis [15]. The ratio of UVA/UVB and absolute UV irradiance depends on many factors such as latitude, season, time of day, and altitude. For example, UV radiation level increases by ∼10% with every 1000 m in altitude [18]. The UV Index, introduced in 1992 and adopted by WHO in 1994, is a useful parameter to characterize solar UV radiation expected for a given day in a specific location. The index (a scale from 1 or “low” to 11 and higher or “extreme”) can be used to predict the risk of harmful effects related to sunlight exposure [19]. It is worth adding that, apart from sunlight, there are other sources of UV radiation that can induce photosensitivity reactions. The artificial sources of UVR are presented in Table 1. Among them are specialized lamps used in phototherapy and dental care as well as tanning beds used in solariums.

Drugs inducing photosensitivity have few specific physicochemical properties, in addition to the ability to absorb UV-vis radiation. They are resonating molecules with a cyclic or tricyclic structure having halogen substituents and heteroatoms. These molecules possess relatively low molecular weight—between 300 Da and 500 Da—and distribute to light-exposed tissues. Photosensitizing drugs absorb a delimited range of electromagnetic radiation including visible and ultraviolet (UV) light (wavelength between 290 nm and 700 nm) [23,24,25]. Their Molar Extinction Coefficient (MEC) is greater than 1000 L mol^−1^ cm^−1^ [13]. Absorption of photons from the electromagnetic spectrum by a photosensitizer leads to promotion of the electron status of the molecule to the state of higher energy content—an excited singlet state [25,26]. The molecule in the excited singlet state is very unstable and it rapidly undergoes internal conversion. The excited photosensitizer may return to the ground state by several pathways including emission of heat or fluorescence, charge transfer, free radicals formation, chemical alteration, or crossing to the triplet excited state [26]. The triplet excited state is much more stable and has a longer lifetime than the singlet excited state. Moreover, the molecule in these states is very reactive and takes part in many photosensitization reactions [27]. Many drugs inducing photosensitivity are photoreactive and they undergo degradation upon exposure to the sunlight. Photodegradation of drugs is usually complex and multidirectional. The major reactions include photoaddition, photocyclization, photodealkylation, photodecarboxylation, photodehalogenation, photodehydrogenation, photodimerization, photoelimination, photoinduced hydrolysis, photoisomerization, photooxidation, photoreduction, and photoinduced ring cleavage [28].

### 3.1. Photoallergy

Photoallergy is an immunologically mediated type of photosensitivity reaction and applies to delayed (cell-mediated) and immediate (humoral-mediated) hypersensitivity responses to a photosensitizing agent [23,26]. Photoallergic reactions are distinguished by (i) no appearance on the first exposure to the photosensitizer, (ii) necessity of an incubation period for the immunologic memory after the first exposure, (iii) cross-reactions between molecularly similar drugs, (iv) the requirement of a low drug dose for a reaction, and v/chemical alteration of photosensitizer and covalent binding with a carrier [25,29,30].

The key event in the pathomechanism of photoallergy is the photobinding of the drug or its metabolite to the carrier protein leading to the formation of a complete photoantigen. The photosensitizer molecule, after UV radiation exposure, can convert to a stable photoproduct and acts as a hapten, which interacts with the protein resulting in immunologically active covalent photoadduct generation. Drug-derived hapten can also be a short-lived intermediate in the unstable excited state. The molecule reverts to the ground state with energy releasing, which facilitates conjugation with a carrier protein [16,30,31].

Photoproduct-protein binding is the result of the interaction between the electrophilic group of photoproducts and the nucleophilic group of amino acid side chains. Amino acids with nucleophilic properties, responsible for the photobinding are lysine (ε-amino group), cysteine (sulfhydryl group), and histidine (imidazole group) [32]. An example of an electrophilic group occurring in the photosensitizer molecules is trifluoromethyl moiety. This group is highly susceptible to nucleophilic substitution after exposure to UV radiation [33,34,35].

The complete photoantigen is recognized and taken up by epidermal Langerhans cells, which are cutaneous dendritic cells [16]. Following photoantigen uptake, Langerhans cells start to produce interleukin (IL)-1β, which affects the migration and maturation of these cells. IL-1β stimulates epidermal keratinocytes to the production of TNF-α promoting migration of Langerhans cells to the skin-draining lymph nodes [36]. In the maturation process, Langerhans cells undergo various phenotypical and functional modifications including: (i) reduction in phagocytic ability, (ii) upregulation of the expression of co-stimulatory cell surface molecules, such as CD86, CD83, CD54, CD40, and the major histocompatibility complex class II (MHC II) molecules, (iii) changes in chemokine receptor profile-downregulation of skin-homing chemokine receptors (CCR1, CCR2, CCR5, and CCR6), and (iv) upregulation of chemokine receptors taking part in the migration (CCR4, CXCR4, and CCR7) [37,38,39,40].

Photoantigen-containing Langerhans cells migrate to the skin-draining lymph nodes, express photoantigen on the cells surface in association with MHC II molecules, and present it to naive T cells. This leads to the activation of T cells and formation of photoantigen-specific, memory T cells. They express the cutaneous lymphocyte antigen involved in homing cells to the dermis [41]. During the next contact with the photoantigen, memory T cells convert to effector T cells secreting interferon-γ (IFN-γ), which induces apoptosis of keratinocytes, promotes migration of macrophages, NK cells, and stimulate degranulation of mastocytes and basophils. All these processes lead to the evolution of inflammatory reactions [40,41,42].

### 3.2. Phototoxicity

Phototoxicity represents direct cellular damage by photoactivated compounds via a nonimmunologic pathway. Typical characteristics of phototoxic reactions are (i) their appearance after the first exposure to a photosensitizer, (ii) their occurrence minutes to hours after sunlight exposure, (iii) necessity of high drug concentration, (iv) a dose-dependent effect, and (v) no cross-reactions between structurally related drugs [25,30].

Phototoxicity can be divided into oxygen-dependent (photodynamic) or oxygen-independent (nonphotodynamic) reactions [16]. In the photodynamic reactions, photosensitizer occurs in the excited triplet state and it reverts to the ground state by electron/hydrogen or energy transfer. An electron or hydrogen transfer results in the production of free radicals, which may interact with ground-state oxygen. This interaction leads to the formation of reactive oxygen species (ROS), such as superoxide anion, hydrogen peroxide, and hydroxyl radical, which are responsible for oxidation damage of the cell. Alternatively, an excited triplet photosensitizer may transfer energy to the oxygen, which results in singlet oxygen generation. This compound is a type of ROS and takes part in the oxidation of cell components [27,43].

Nonphotodynamic reactions refer to direct cellular damage by excited photosensitizers in deoxygenated conditions [16]. The first type of these reactions is covalent binding between an excited drug or metabolite and cellular macromolecules [44]. Photosensitizers may also interact with cell constituents by electron or hydrogen atoms transfer [45]. In another pathway, an excited photosensitizer could undergo decomposition, and obtained photoproducts may react with cell components or act as a new photosensitizer [27].

Subcellular targets of excited photosensitizers depend on their lipid solubility. Photosensitizers with hydrophilic features cause membrane injury, whereas lipophilic compounds diffuse into the cell and damage intracellular components, such as mitochondria, lysosomes, and the nucleus [16,25].

Drug/metabolite—cellular macromolecules—adducts and damage of cell constituents lead to the release of erythrogenic mediators. Biologically active agents that participate in the phototoxicity process are eicosanoids, histamine, complement, and proteases [9,44,46].

### 3.3. Biochemical Effects of Oxidative Stress

Phototoxicity is associated with ROS generation at the cellular level. In turn, oxidative stress contributes to disturbances in cell homeostasis and induction of an inflammatory reaction that results in the development of typical skin lesions. Clinical manifestations of phototoxicity include exaggerated sunburn with itching and burning sensations. Histologically, necrotic epidermal keratinocytes and dermal infiltration of neutrophils and lymphocytes occur [29,30].

Overproduction of ROS causes oxidative damage of lipids, proteins, and nucleic acids [27]. DNA defects lead to upregulation of p53, which is connected with cell cycle arrest, inhibition of cell proliferation, or induction of skin cell apoptosis [47,48]. Moreover, p53 protein increases the synthesis of tyrosinase, a key enzyme of melanogenesis. Therefore, it contributes to the enhancement of melanin synthesis that may result in hyperpigmentation [49]. ROS-induced DNA damage may include loss of function mutations in the p53 gene, which in turn leads to excessive cell proliferation and photocarcinogenesis [47,48].

Oxidative stress affects the release of inflammatory mediators. It has been demonstrated that ROS activates JNKs (c-Jun amino-terminal kinases) and p38 pathways. These kinases support the production of chemoattractants, cytokines, chemokines leading to chemotaxis and activation of helper T lymphocytes, and macrophages as well as neutrophils [50,51]. Moreover, ROS-dependent inhibition of IκBa protein causes NF-kB activation. This molecule influences the transcription of many factors, among others, cytokines, chemokines, cyclooxygenase 2, and nitric oxide synthase [52]. Cyclooxygenase 2 catalyzes the formation of prostaglandins, thromboxane, and prostacyclin. It has been reported that prostaglandin e2, as well as nitric oxide, mediates vasodilatation that may be the reason for erythema and edema, observed in clinical presentation [53]. Moreover, these factors augment melanogenesis leading to hyperpigmentation [54]. Generated nitric oxide may also interact with ROS to form peroxynitrite, a very reactive molecule causing oxidative damage of cellular components [50].

### 3.4. Role of Melanin Biopolymers in Photosensitization

Skin pigmentation is the main protective factor against the harmful effects of UV radiation. The color of the skin, hair, and iris of the eye is mainly determined by macromolecular dyes called melanin. The pigment is the end-product of melanogenesis, the multistep transformation of tyrosine, occurring in specialized organelles of melanocytes. Melanin is transferred from melanocytes to adjacent keratinocytes and deposited within skin cells [55]. The protective role of melanin is due to its ability to disperse and absorb UV radiation. Absorption of sunlight by melanin is greatest in the short-wave part of UV radiation and gradually decreases when it passes towards the visible light. It is estimated that melanin can absorb 50–75% of UV radiation [56,57]. It is believed that the most important role of melanin is to protect genetic material. This is confirmed by the location of melanosomes in skin cells where melanin supranuclear caps are observed above the nucleus [55].

Mammalian melanocytes synthesize two types of melanin, i.e., eumelanin and pheomelanin. Although both types can absorb light in the UV and visible ranges, they differ in their physiochemical properties significantly. Black-brown eumelanin is a nitrogenous polymer composed of 5,6-dihydroxyindole (DHI) and 5,6-dihydroxyindole-2-carboxylic acid (DHICA) subunits at different oxidation degrees (5,6-quinone, semiquinone, and 5,6-dihydroxy units) [14,58]. The chemical structure and physicochemical properties of eumelanin cause it to act as a great photoprotector and a physiological redox buffer, with both reducing and oxidizing capacities, and neutralize free radicals [59,60]. It was shown that the antioxidant properties of melanin are related to the superoxide dismutase-like activity [61]. Moreover, the biopolymer converts absorbed photon energy into heat [62]. This nonradiative relaxation process is highly efficient and reduces cellular damage. In contrast, yellow-to-reddish brown pheomelanin is sulfur and nitrogen-containing polymer. The presence of 1,4-benzothiazine subunits causes pheomelanin to act as a photosensitizer. After absorption of UV-visible light, the pheomelanin chromophore goes to an excited state and returns to the ground state by energy or electron/hydrogen transfer. This process is the source of harmful reactive oxygen species, i.e., singlet oxygen, hydrogen peroxide, superoxide anions, or hydroxyl radicals, which damage, directly or indirectly, cell structures as well as disrupt their functions [14,63,64].

Melanin synthesis in vivo leads to the production of a mixture of pheo- and eumelanins. For this reason, “mixed melanogenesis” is observed [65,66]. Thus, the final photoprotective effect depends on the total amount of melanin as well as the proportion of melanin types. Differences in pigmentation and predisposition to sunburn are the basis for the classification of races (Celtic, Caucasian, and Negroid race) and skin phototypes according to the Fitzpatrick scale (I–VI phototype) [67]. The photoprotective role of melanin is confirmed by epidemiological data indicating that the incidence of skin cancers, including melanoma, is inversely proportional to the degree of pigmentation [68].

Considering the role of melanin in the aspect of drug phototoxicity, the drug-melanin complexes should be mentioned. Melanin pigments, both eumelanin and pheomelanin, bind drugs, which affects their efficacy and toxicity as well as the occurrence of side effects. The results of previously conducted studies show that many phototoxic drugs form complexes with melanin polymers [69]. The percentage of a drug bonded to melanin and the kinetics of drug-melanin complexes formation as well as binding parameters were determined for many phototoxic drugs, including fluoroquinolone antibiotics, tetracyclines, and nonsteroidal anti-inflammatory drugs [70,71,72]. Compound binding to tissue components, e.g., melanin, is one mechanism by which drug retention and/or accumulation can occur. Thus, melanin binding leads to an increase in tissue levels of that compound. The process also increases the risk of the drug-induced cytotoxic effect. It was found that melanin-producing cells are sensitive to the phototoxic drug, also without exposure to UV radiation [70,72,73,74]. The tested drugs inhibited human melanocyte proliferation and induced changes in melanin synthesis and in the activity of antioxidant enzymes. Our preliminary study of minocycline revealed that the drug significantly increased melanin content and the activity of tyrosinase—the key enzyme of melanogenesis. Besides, it triggered the supranuclear accumulation of tyrosinase, similar to UVA and UVB radiation [75,76].

## 4. Characteristics of Drugs with Photosensitive Potential

### 4.1. Nonsteroidal Anti-Inflammatory Drugs

Nonsteroidal anti-inflammatory drugs (NSAIDs) are the most widely used drugs worldwide. They account for 5–10% of all medications prescribed each year. Moreover, several drugs of these groups are active components of over-the-counter preparations [77].

NSAIDs are a chemically highly heterogeneous class of compounds that cause many cutaneous reactions, such as urticaria, Stevens-Johnson syndrome, lichenoid eruptions, and photosensitization. These side effects may be caused by oral as well as topical formulations of drugs, however, they are more frequent for topical drug administration [30,78].

NSAIDs are responsible for phototoxic and photoallergic reactions. The following drugs have phototoxic potential [16]:

(a) arylpropionic acid analogs: benoxaprofen, ibuprofen, ketoprofen, carprofen, nabumetone, tiaprofenic acid, naproxen;
(b) salicylic acid derivatives: aspirin;
(c) anthranilic acid analogs: meclofenamic acid;
(d) pyrazolidinedione derivatives: oxyphenbutazone, phenylbutazone.

Benoxaprofen was removed from the European market in 1982 because of its acute phototoxic effects [27]. For ibuprofen, it has been demonstrated that it causes dose-dependent phototoxic hemolysis after UVA irradiation [78]. Ketoprofen irradiation induces cellular lipids peroxidation and damage of DNA and cell membranes [79].

Photoallergic reactions are caused by benzydamine hydrochloride, piroxicam, and topical tiaprofenic acid, suprofen, ketoprofen, diclofenac [16,29].

Benzydamine is used for the treatment of stomatitis and vaginal/rectal mucositis. After UVA radiation exposure, benzydamine causes photoallergic contact dermatitis on the face, neck, forearms, dorsum of hands, and the “V” area of the upper chest. Orally, application of benzydamine can lead to cheilitis [80,81].

Piroxicam causes systemic photoallergy manifested by dyshidrosis, acute eczema on the face, scattered erythematous papules, and vesicles on the face and dorsum of the hands. Piroxicam cross-reacts with thimerosal. The photoallergic reactions may be due to photoproducts of the piroxicam [29,82,83,84].

Topical diclofenac induces erythematous and scaly plaque with vesicles and yellow crusting [85]. There were demonstrated cross-reactions between diclofenac and topical aceclofenac [29].

Ketoprofen is the most frequent cause of photoallergy due to topical NSAIDs [86]. Clinical manifestations of photoallergy to ketoprofen include erythema, edema, and papulovesicular, extending beyond the areas of drug and sun exposure [87]. There were demonstrated cross-sensitivity reactions between ketoprofen and tiaprofenic acid, ibuprofen, suprofen, fenofibrate, and sunscreens containing oxybenzone [79,88].

#### Ketoprofen

**Figure 1:**
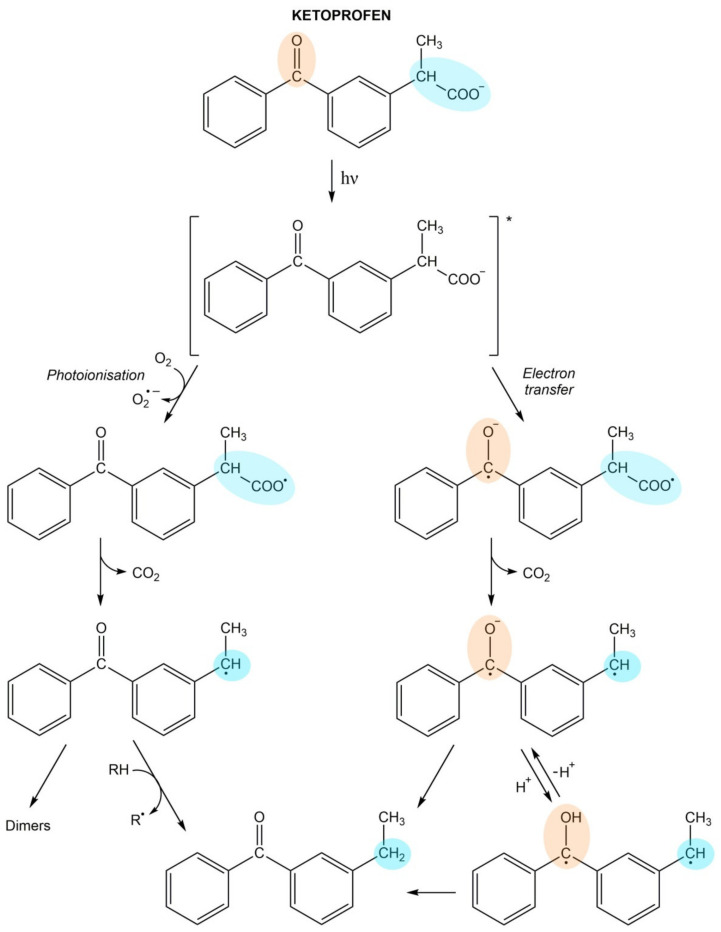
Mechanism of ketoprofen photodecarboxylation process.

During UV irradiation ketoprofen undergoes the photodecarboxylation process, which occurs through two mechanisms—photoionization and intramolecular electron transfer (Figure 1). In the first pathway, the ketoprofen molecule in the excited state ejects an electron, which is scavenged by oxygen [79,89]. After this, the ketoprofen radical undergoes decarboxylation leading to the generation of benzylic radicals, which may form dimers [90]. In the alternative mechanism, the electron is transferred from the carboxyl (donor) to the carbonyl (acceptor) groups of the ketoprofen molecule in the triplet state [79]. This leads to the formation of a biradical triplet. Following the release of carbon dioxide, a biradical triplet is converted to a benzylic carbanion. It achieves protonation equilibrium between carbanion and biradical forms. The final product of this pathway is 3-benzoylphenylethane generated by an intramolecular H-shif [89].

**Figure 2:**
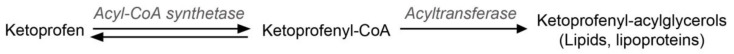
Synthesis of ketoprofenyl-acylglycerols.

Ketoprofen-induced photosensitive dermatitis results from long-term retention and high drug levels in the skin. Ketoprofen is recognized as a fatty acid substrate by acyl-CoA synthetase and it leads to the ketoprofenyl-CoA formation (Figure 2). Subsequently, ketoprofenyl-CoA may be used for acylglycerols synthesis catalyzed by acyltransferase. Therefore, ketoprofen is present in adipose tissue for a long time [91].

Ketoprofen causes photoadducts formation that leads to a photoallergic reaction. Shinoda et al. [92] reported that ketoprofen reacts with amino acids. However, Karlsson et al. [86] showed that ketoprofen induces the generation of amino acid photoadducts, and photoallergy may be caused by an immunogenic complex not containing ketoprofen moiety.

It has been reported that ketoprofen under UVA irradiation significantly affects the viability and function of Langerhans cells and keratinocytes. Exposure to ketoprofen and UVA radiation results in morphological changes of epidermal Langerhans cells and an increase in the expression of surface molecules—MHC class II, CD86, CD80, CD54, and CD40—on these cells. Moreover, the treatment of keratinocytes with ketoprofen and UVA enhances the production of IL-1α and GM-CSF that upregulate the function of Langerhans cells [93].

Ketoprofen phototoxicity has been assessed by in vitro methods. The mechanism of ketoprofen phototoxicity includes singlet oxygen production that leads to single and double-strand DNA breakage, arrest at G2/M phase of the cell cycle, and apoptosis [94]. Moreover, irradiation of ketoprofen causes photohaemolysis, lipid peroxidation, and DNA damage involving pyrimidine dimers formation [79].

### 4.2. Cardiovascular Drugs

Photosensitivity is a side effect of the following classes of cardiovascular drugs:

(a) diuretics: hydrochlorothiazide, chlorthalidone, indapamide, bumetanide, furosemide;
(b) angiotensin-converting enzyme (ACE) inhibitors: ramipril, quinapril, enalapril;
(c) angiotensin receptor blockers (ARBs): valsartan;
(d) calcium channel blockers: amlodipine, nifedipine, diltiazem;
(e) beta-blockers: tilisolol;
(f) antiarrhythmic drugs: amiodarone;
(g) antithrombotic agents: clopidogrel, triflusal;
(h) others: rilmenidine, methyldopa.

Sulphonamide-derived diuretics have been described as inducing photosensitivity. It is estimated that between 1 and 100 per 100,000 patients treated with thiazide diuretics exhibit photosensitivity [95,96]. The thiazide class includes, e.g., hydrochlorothiazide and chlorthalidone. It has been reported that thiazides induce pseudoporphyria [97]. Moreover, hydrochlorothiazide use has been associated with the following symptoms of photosensitivity: cutaneous lupus erythematosus [98,99], photoleukomelanoderma [100], photoonycholysis [101], lichenoid photosensitivity [102], cheilitis, erythema, and eczema [96]. Pseudoporphyria has been reported for furosemide and bumetanide, belonging to the loop diuretics [103]. Indapamide, a thiazide-like diuretic, has been shown to induce photoonycholysis [104].

Photosensitivity reactions have been described for ACE inhibitors without the thiol group. They cause a photosensitive lichenoid eruption and erythematous rash with generalized pruritus [105,106].

Valsartan is an ARB that induces photosensitivity manifested by a pruritic rash on light-exposed areas [107].

It was reported that amlodipine and nifedipine induce photodistributed facial telangiectasia [108,109]. Diltiazem causes photoallergic dermatitis that manifests as photodistributed hyperpigmentation. The etiopathogenesis of diltiazem photosensitivity is unknown [110,111,112].

Tilisolol is a beta-blocker that causes a photoallergic reaction after exposure to UVA radiation [113].

Phototoxicity affects 25–75% of patients treated with amiodarone [114]. Clinical manifestations associated with phototoxicity of amiodarone include a burning sensation, erythema, and edema of the face, neck, and hands following a few minutes after sun exposure [115,116]. Skin reactions reported by patients also include a fine maculopapular rash and a slate-grey discoloration of sun-exposed areas. Blue-grey pigmentation occurs in patients undergoing long-term therapy [116,117]. Amiodarone use is also connected with retinal phototoxicity [118]. Amiodarone and its metabolite, desethylamiodarone, have photosensitizer properties [8,115].

Photosensitivity induced by triflusal may manifest as erythematous and exfoliative lesions, ectropion, loss of skin folds, and hyperpigmentation [119]. Clopidogrel use is connected with lichenoid eruption [120].

Rilmenidine stimulates central imidazoline receptors I1. It has been demonstrated that the drug induces phototoxic reactions after exposure to UVA radiation. Clinical manifestation is erythema with a burning sensation and pruritus on sun-exposed areas [121]. Methyldopa is a centrally acting antihypertensive that decreases catecholamines synthesis [8]. Photosensitivity to methyldopa has been reported. Methyldopa causes erythematous pruritic papulovesicular eruption on light-exposed areas [122].

#### 4.2.1. Thiazides

Thiazides have phototoxic potential and the trigger factor is UVA radiation. The mechanism of thiazides phototoxicity includes lipid peroxidation and extension DNA damage induced by UVA radiation [123]. It has been reported that hydrochlorothiazide enhances the UVA-induced formation of cyclobutane pyrimidine dimers [124]. It has been shown that the main product of hydrochlorothiazide photolysis is ethoxyhydrochlorothiazide, generated by photosubstitution of chloride by the ethoxy group [125].

#### 4.2.2. Calcium Channel Blockers

**Figure 3:**
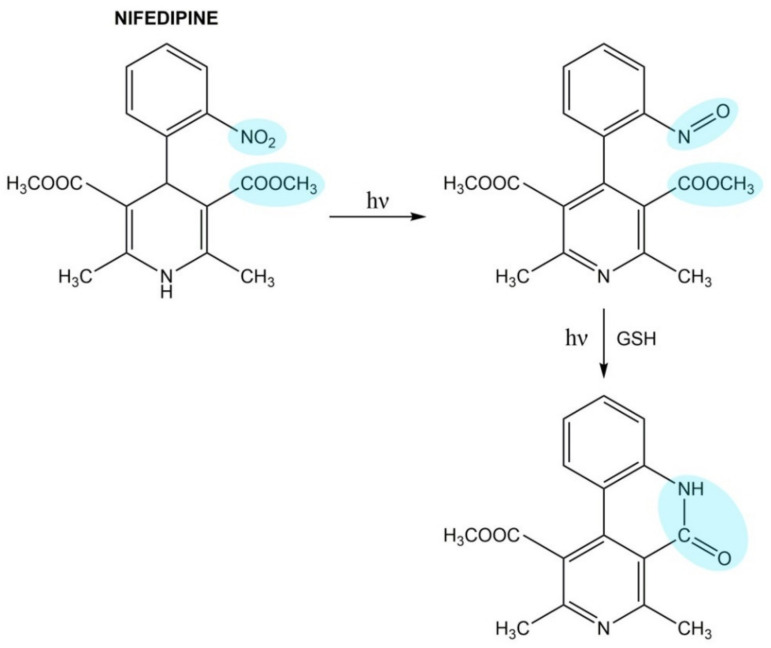
The photodegradation process of nifedipine.

Amlodipine and nifedipine absorb UVA radiation that results in ROS generation [126]. The photodegradation process of nifedipine has been shown (Figure 3). Exposure to UVA radiation results in the photooxidation of nifedipine to nitroso-photoproduct. The product of the reaction is 4-(2′-nitrosophenyl)-pyridine, which is converted to lactam derivate in the presence of glutathione [127,128].

#### 4.2.3. Amiodarone

**Figure 4:**
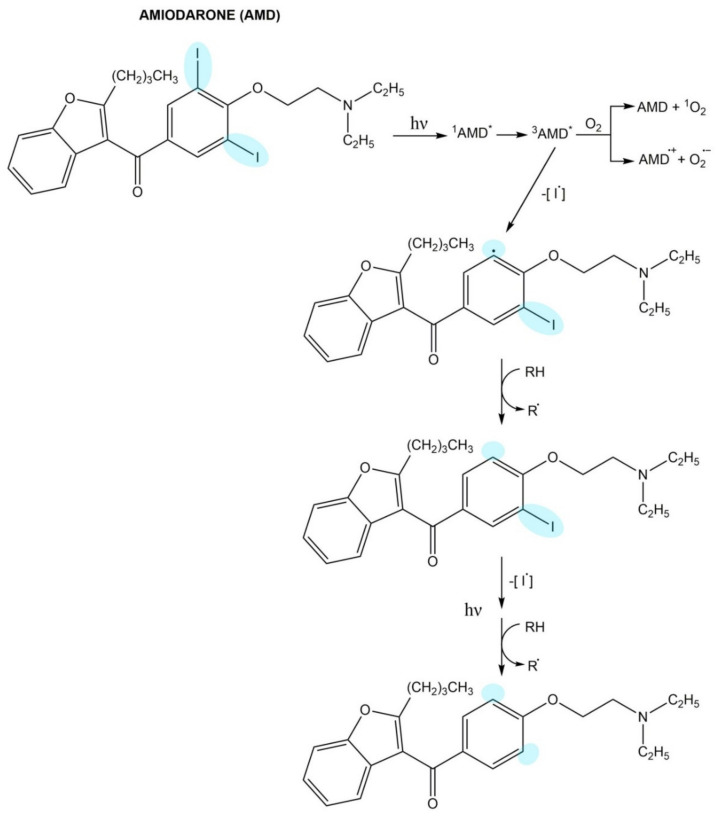
The photodegradation process of amiodarone.

Amiodarone and its main metabolite, desethylamiodarone, undergo photodegradation in a similar way (Figure 4). UV irradiation leads to the crossing of the molecule to the singlet excited state and then to the triplet excited state. Amiodarone/desethylamiodarone in the triplet excited state may interact with oxygen or may be converted to photoproducts. Electron transfer from the excited molecule to oxygen results in superoxide generation. In turn, the photoproducts formation includes the following stages: photodehalogenation, homolytic cleavage of the C-l bond, aryl radical formation, and hydrogen abstraction [129].

### 4.3. Antihyperlipidemic Drugs

Statins, one of the most commonly used drugs worldwide, have been associated with photosensitivity reactions [8]. It has been reported that atorvastatin causes phototoxicity manifested by edematous actinic erythema on sun-exposed sites [130]. Simvastatin and pravastatin were shown to induce photodistributed erythema multiforme [131]. Moreover, simvastatin use is connected with chronic actinic dermatitis [132,133].

Among the fibric acid derivates, fenofibrate has the highest photoallergy potential. Clinical manifestations of fenofibrate-induced photoallergy include erythematous, lichenoid, eczematous, or papulovesicular eruption on sun-exposed areas [134,135].

#### Fenofibrate

**Figure 5:**
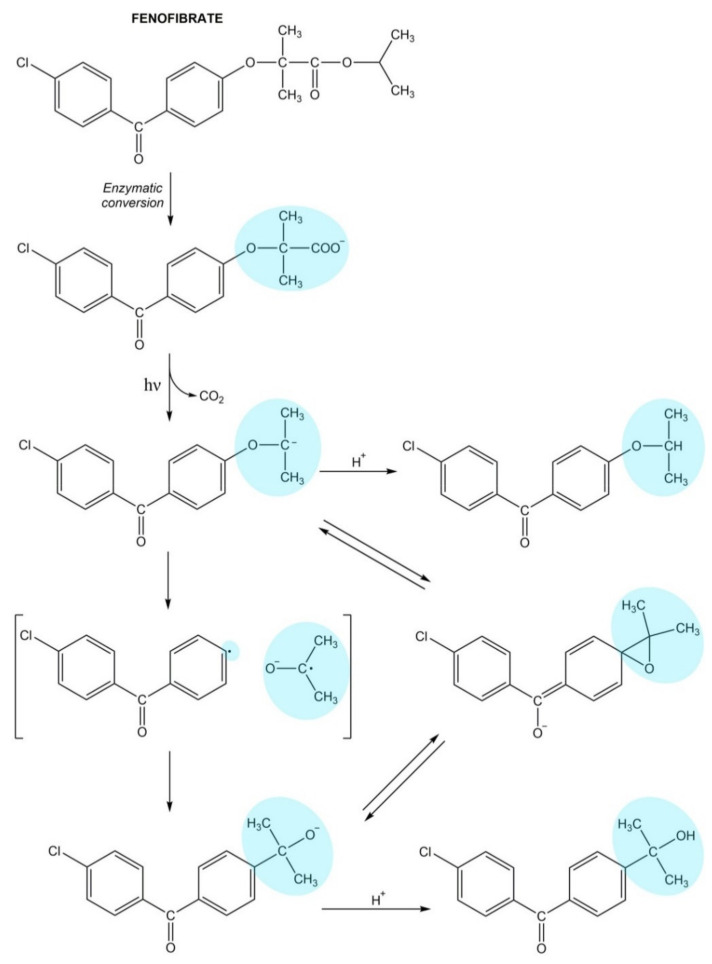
The photodecarboxylation process of fenofibrate.

Fenofibrate is an inactive prodrug that is rapidly hydrolyzed to the active metabolite-free fenofibric acid after oral administration. Free fenofibric acid has high photochemical reactivity because of the benzophenone chromophore. Fenofibric acid molecule absorbs UVA radiation and undergoes photodecarboxylation resulting in the formation of photoproducts 4-chloro-4′-isopropoxybenzophenone and 4-chloro-4′-(1-hydroxy- 1-methylethyl)benzophenone (Figure 5) [129,136].

Photoproducts interact with proteins that lead to photoadducts generation and photoallergic reaction. The excited benzophenone chromophore accepts hydrogen atoms from an amino acid residue of biomolecules and this results in the formation of covalent photoadducts [137]. It has been reported that fenofibric acid stimulates prostaglandin production by fibroblasts and keratinocytes exposed to UVA radiation [138].

Fenofibrate has phototoxicity potential also. It has been shown that fenofibrate causes photohaemolysis, lipid photoperoxidation, and DNA damage [129,139,140].

### 4.4. Psychotropic Drugs

The following psychotropic drugs have photosensitivity potential:

(a) antipsychotics: chlorpromazine, fluphenazine, perphenazine, thioridazine, chlorproethazine, flupenthixol, olanzapine, aripiprazole, clozapine;
(b) antidepressants: imipramine, clomipramine, paroxetine, fluvoxamine, fluoxetine, citalopram, sertraline, escitalopram, phenelzine, venlafaxine;
(c) anxiolytics: alprazolam, chlordiazepoxide.

Phenothiazine antipsychotics induce phototoxic and photoallergic reactions. UVA radiation is a trigger factor of phenothiazines photosensitivity [16,141]. Photosensitivity cases for the following phenothiazines were reported: chlorpromazine, chlorproethazine, perphenazine, fluphenazine, cyamemazine, and thioridazine. Clinical manifestations include eczematous or hyperpigmented papular eruption in sun-exposed sites [142,143,144,145,146,147].

Flupenthixol is a thioxanthene derivate that causes photosensitivity reactions because of chemical similarity to the phenothiazines. Flupenthixol use is connected with erythematous, eczematous eruption [148].

Atypical antipsychotics also induce photosensitivity. It has been reported that olanzapine and aripiprazole cause photoonycholysis [149]. Clozapine was shown to induce erythematous eruption with tense skin blisters [8].

Photosensitivity was demonstrated for the following antidepressant groups: tricyclic antidepressants, selective serotonin reuptake inhibitors (SSRIs), serotonin-noradrenaline reuptake inhibitors (SNRI), and monoamine oxidase inhibitors [8].

Tricyclic antidepressants with photosensitivity potential are imipramine and clomipramine. Imipramine causes purple/slate-grey/golden-brown/dark brown discoloration of the sun-exposed areas and iris color change. There is a hypothesis that imipramine switches melanogenesis towards pheomelanin [150]. Clomipramine was shown to induce photoallergy [8].

Photoreaction cases have been reported for the following SSRIs: paroxetine, fluvoxamine, fluoxetine, citalopram, sertraline, and escitalopram. Clinical manifestations of SSRIs photosensitivity are exanthematous pustulosis [151], eczematous eruption [152], diffuse erythema and edema [153], brownish-black pigmentation [154], and itching erythema with infiltrations and blisters [155,156].

The monoamine oxidase inhibitor phenelzine and SNRI venlafaxine were shown to induce photosensitivity [8]. It has been reported that venlafaxine causes photodistributed eruptive telangiectasia [157].

The anxiolytics inducing photosensitivity are alprazolam and chlordiazepoxide. These drugs’ use are connected with pruritic erythema and eczematous reaction in sun-exposed sites [8,158].

#### Phenothiazines

**Figure 6:**
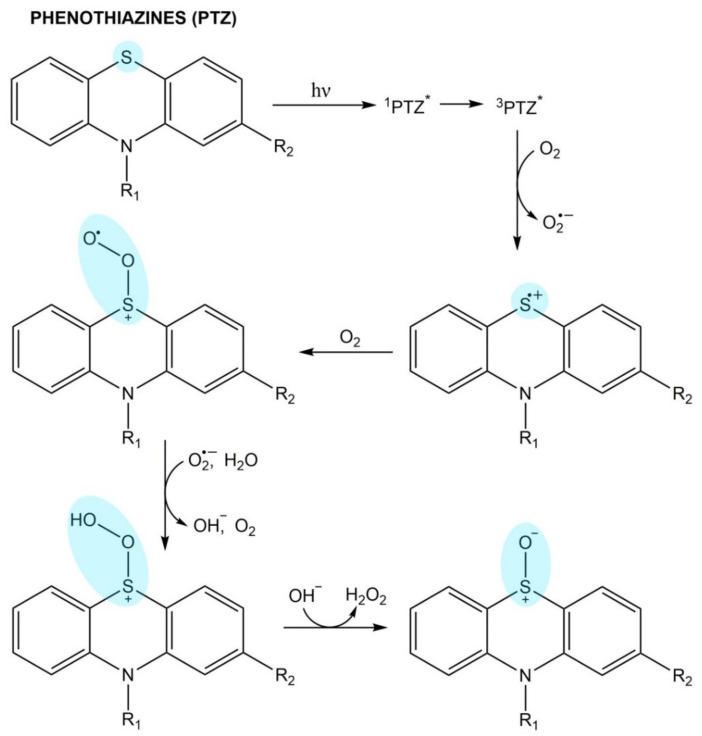
Generation of sulfur-centered peroxy radical in photolysis of phenothiazines.

Phenothiazines absorb UV radiation and undergo photolysis including the formation of carbon-centered radicals and cation radicals [159]. The drug molecule in triplet excited state or cation radicals may interact with molecular oxygen that results in singlet oxygen generation (Figure 6). Moreover, reaction with oxygen leads to the production of oxidized products [160,161]. It has been reported that 2-chloroderivatives undergo dechlorination and then photoreduction or substitution. The photoproducts of trifluoromethyl derivatives are carboxylic acids [141].

The phenothiazine radicals may interact with biomolecules leading to photoantigen formation and photoallergy induction [159]. Phenothiazines cause phototoxic cellular damage through DNA strand breaks, lipid peroxidation, and an increase in protein photoaggregation. The phototoxic potential of phenothiazines was ranked as follows: fluorinated derivates > chlorinated derivates > nonhalogenated derivates [162,163,164,165].

### 4.5. Antiretrovirals

It has been reported that simeprevir causes pseudoporphyria cutanea tarda through a phototoxic mechanism. Clinical manifestations are erythematous scaly patches, bullae, painful blisters, and erosions on the sun-exposed areas [166]. Tenofovir is connected with **a** photoallergic reaction manifested as erythema and hyperpigmented plaques [167]. Efavirenz also was shown to induce photoallergy and causes pruritic edematous erythema [168,169,170,171].

### 4.6. Antifungals

Azole antifungals are connected with photosensitivity reactions. Azoles are subdivided into two groups—imidazoles and triazoles [172]. The imidazole antifungal causing photoallergy is ketoconazole. The topical use of ketoconazole and exposure to UVA radiation induce photoallergic contact dermatitis [173]. The triazoles shown to cause photosensitivity are itraconazole and voriconazole. Itraconazole induces an erythematous plaque with papules and vesicles on sun-exposed areas [174]. Voriconazole use is connected with phototoxic reactions. Bullous photodermatosis [174,175] and photodistributed erythema have been reported during voriconazole treatment [176]. Moreover, chronic voriconazole therapy is associated with accelerated photoaging [177] and photocarcinogenesis-melanoma [178], and squamous cell carcinoma development [179,180,181].

Another synthetic antifungal-terbinafine, an allylamine derivate, is shown to induce subacute cutaneous lupus erythematosus. Clinical manifestations are erythematous, and scaling plaques with hyperpigmented borders [182].

#### Voriconazole

**Figure 7:**
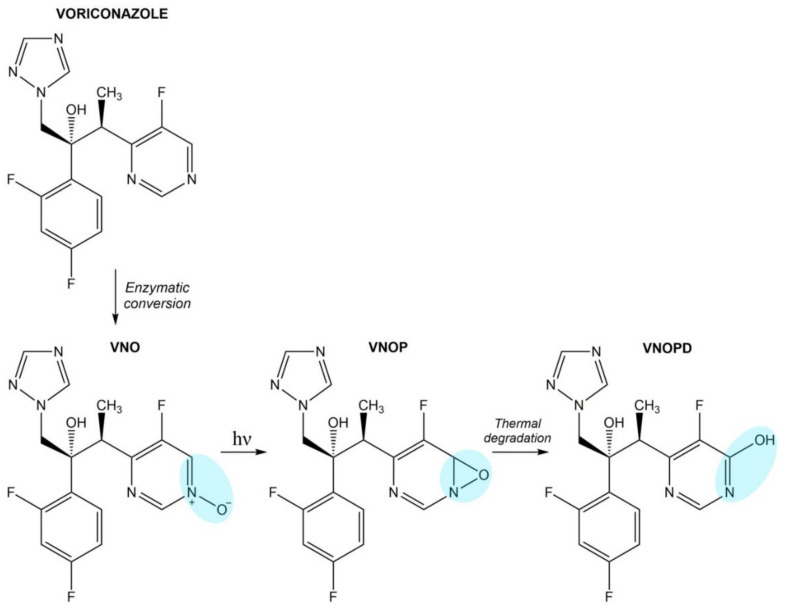
The photodecarboxylation process of voriconazole metabolite.

Voriconazole itself possesses weak UV absorbance, but its major metabolite, voriconazole N-oxide (VNO), shows a prominent absorbance in the UV spectral region (Figure 7). Voriconazole N-oxide is converted upon UVB radiation to photoproduct (VNOP), which undergoes transformation to stable VNOPD having phototoxic potential [183].

It has been reported that voriconazole N-oxide and its photoproduct sensitize keratinocytes to UVA radiation and this leads to cellular damage through reactive oxygen species formation and oxidative DNA damage [184].

### 4.7. Antibacterial Drugs

The following groups of antibacterial drugs are connected with photosensitivity induction: tetracyclines, fluoroquinolones, β-lactams, antituberculous agents, and sulfonamide derivatives.

The tetracyclines cause phototoxic cutaneous reactions. These drugs have a broad absorption spectrum over a range of UVA wavelengths [185]. It has been reported that doxycycline causes a burning, tingling rash, erythema, lichenoid eruption on sun-exposed areas, and photoonycholysis [8,186,187]. Tetracycline is associated with solar urticaria, pseudoporphyria, and photoonycholysis [8,188].

The fluoroquinolones cause phototoxic and photoallergic reactions. The use of these antimicrobial drugs is connected with photocarcinogenesis also [29]. Usually, under UVA irradiation, fluoroquinolones cause phototoxicity and phototoxic potential differs between derivates. The significant phototoxic outcomes have analogs with a C-8-fluorine/chlorine substituent, such as lomefloxacin, sparfloxacin, and clinafloxacin [189]. The fluoroquinolone ranking from the least to the greatest phototoxic potential is as follows: moxifloxacin < gatifloxacin < trovafloxacin < ofloxacin < norfloxacin < levofloxacin < gemifloxacin < grepafloxacin < ciprofloxacin < ulifloxacin < pefloxacin < enoxacin < sparfloxacin < clinafloxacin < fleroxacin < lomefloxacin [29]. Clinal manifestations of fluoroquinolones phototoxicity are bullous eruptions, pseudoporphyria, and porphyria cutanea tarda [26].

β-lactams connected with photosensitivity are ceftazidime and cefotaxime. Ceftazidime increases susceptibility to sunburn and cefotaxime causes photodistributed telangiectasia [8,190].

Antituberculous agents inducing photosensitivity are isoniazid and pyrazinamide. Isoniazid use is connected with photosensitive lichenoid eruptions [191]. Pyrazinamide causes erythematous maculopapular rashes with generalized edema on sun-exposed areas [192].

Among the sulfonamide derivates, photosensitivity reactions have been reported for sulfamethoxazole, dapsone, and sulfasalazine [29]. Dapsone induces erythematosus, itchy, papulovesicular lesions over sun-exposed areas [193,194]. Sulfasalazine use is shown to cause diffuse hyperpigmentation [26].

#### 4.7.1. Fluroquinolones

**Figure 8:**
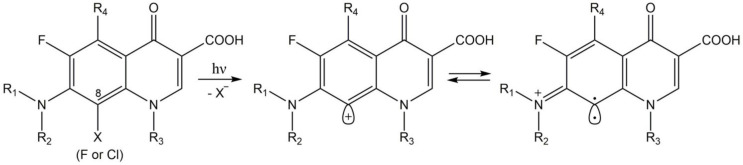
The photolysis of 8-halogenated fluoroquinolones.

Fluroquinolones absorb UVA radiation. It has been shown that the halogen atom at position 8 affects on high photolability of the drug (Figure 8). Under UVA irradiation, the fluoroquinolone molecule undergoes selective heterolytic photodehalogenation from position 8 that leads to aryl cation formation. Aryl cations possess **a** carbene character; therefore, they are very reactive [195,196]. It has been shown that aryl cations may react with oxygen that results in quinone-imine and hydrogen peroxide production. Hydrogen peroxide is a substrate in hydroxyl radicals formation via Fenton chemistry [197]. The mechanism of fluoroquinolones phototoxicity is not correlated with singlet oxygen generation [198].

It has been reported that reactive oxygen species generated from fluoroquinolones under UVA radiation activate protein kinase C and tyrosine kinase. This leads to activation of phospholipase A2 and cyclooxygenase that stimulate the production of prostaglandins-inflammatory mediators. The release of prostaglandins from dermal fibroblasts induces skin inflammation [199].

The mechanisms of fluoroquinolones phototoxicity include: lipid peroxidation, proteins photooxidation, a decrease in mitochondrial potential, DNA strand breaks, and alterations in the antioxidant defense system [200,201,202].

It has been shown that lomefloxacin causes phototoxicity and photoallergy. Lomefloxacin interacts with proteins and amino acids that result in covalent photoadducts formation. There are two postulated mechanisms for lomefloxacin photobinding to proteins. In the first pathway, high reactive aryl cation direct reacts with amino acids. In the alternative mechanism, electron transfer between the singlet excited state of lomefloxacin and protein leads to photobinding [203].

#### 4.7.2. Tetracyclines

Skin discoloration and photosensitization are the most characteristic side effects of tetracycline antibiotics. Taking into account the type of photosensitization mechanism, it may be stated that tetracycline antibiotics show only phototoxic activity [23]. The photosensitizing properties of tetracyclines result mainly from the structure of the molecule core-partially hydrogenated naphthacene, as well as the presence of a high electron density area in the lower peripheral region of the molecule [204]. Such a structure easily absorbs electromagnetic radiation, which leads to the excitation of the molecule and the transition from the ground state to a higher energy level, singlet excited state. After the excitation, the tetracycline molecule is deactivated by fluorescence and returns to the ground state. Additionally, it may be turned into the triplet state by intercombination transition [27]. Individual tetracyclines differ in the frequency and the degree of intensity of phototoxic reactions. It has been found that phototoxic reactions occur most frequently with the use of demeclocycline, considered the strongest photosensitizer in this group, and doxycycline [30,185]. The frequency of phototoxic reactions during therapy with demeclocycline varies, depending on the daily dose, between 25% and 90%. In the case of doxycycline, phototoxic reactions are observed in 3–42% of patients [8,26].

**Figure 9:**
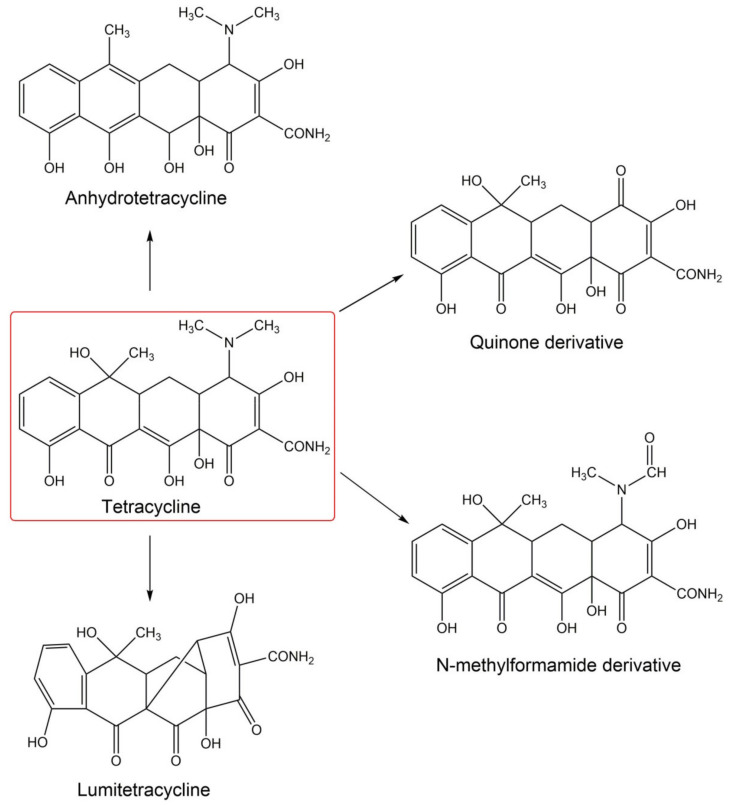
The photolysis of tetracyclines.

The phototoxic effect of tetracyclines is caused by transferring energy from an excited compound to an oxygen molecule. The process leads to the production of singlet oxygen and/or the formation of harmful free radicals [27,205]. UVA radiation and reactive oxygen species can also decompose tetracycline antibiotics into various photoproducts: anhydrotetracyclines, lumitetracyclines, quinones, or N-methylformamide derivatives (Figure 9) [205,206,207,208]. Photodegradation of tetracyclines includes demethylation, hydroxylation, deamination, or dehalogenation [208,209]. The photoproducts are devoid of antibiotic activity and most of them are more toxic than the parent compounds [209,210,211]. The clinical symptoms of tetracycline phototoxicity relate primarily to surfaces exposed to light and include erythema, edema, blisters, urticaria, rash, and separation of the nail plate, onycholysis, and discoloration [212,213].

Skin hyperpigmentation is one of the symptoms of drug-induced phototoxicity. The effect is caused by, among others, the intensification of melanin synthesis, the formation of stable drug-melanin complexes, and drug accumulation in skin cells [214]. The accumulation of tetracyclines in pigmented tissues increases the risk of side effects. To date, the binding of doxycycline, oxytetracycline, and chlortetracycline to melanin polymers has been demonstrated [72,215]. The studies show that the amount of tetracyclines bound to melanin increases proportionally to their concentration and incubation time. The equilibrium state of the complex formation is estimated to be 24 h. Moreover, several studies indicate the cytotoxic and phototoxic potential of tetracyclines to human normal epidermal melanocytes [72,73,216,217]. It has been shown that the simultaneous exposure of melanocytes to tetracycline antibiotics and UVA radiation leads to a decrease in cell viability. The effect has appeared to be proportional to the concentration of the tested drug and the dose of UVA radiation. On the basis of the IC_50_ value, it can be concluded that the cytotoxicity of tetracyclines towards melanocytes decreases in order: doxycycline > tetracycline > oxytetracycline = chlortetracycline. On the other hand, the order of tetracycline phototoxic potential towards melanocytes decreases as follows: chlortetracycline > oxytetracycline > tetracycline > doxycycline. This comparison considers the viability of melanocytes exposed to tetracyclines in concentrations equal to the IC_50_ and 30-min UVA irradiation. The observed differences between the cyto- and phototoxic potential of individual tetracyclines in relation to pigmented cells result, inter alia, from different parameters of the binding to melanin, as well as differences in the structure and physicochemical properties of the tested antibiotics. The high phototoxicity of chlortetracycline may be related to the presence of a chlorine substituent in the 7-position, which is one of the factors increasing phototoxic potential. On the other hand, the lower phototoxicity of doxycycline may result from the absence of a hydroxyl group in the 6-position. This group, present in the structure of the first generation tetracyclines, participates in photolysis of tetracyclines and in the formation of toxic photoproducts, anhydrotetracyclines.

The studies on melanocytes also indicate that tetracyclines, proportionally to the concentration, intensify the effect of UVA radiation on melanogenesis and the activity of antioxidant enzymes (SOD, CAT, GPx) in melanocytes. The observed effect is most likely related to the phototoxic effect of the studied drugs and the increased production of free radicals, mainly reactive oxygen species. Thus, the ability of tetracyclines to form complexes with melanin as well as the cyto- and phototoxic effects on epidermal melanocytes may be one of the reasons for the occurrence of skin adverse effects during therapy.

### 4.8. Antimalarials

Quinidine induces photoallergic contact dermatitis manifested by eczematous plaques, papulovesicular lesions, and cheilitis [218,219]. Hydroxychloroquine has been reported to cause photoallergic and phototoxic reactions. Clinical manifestations are itchy, erythematous, papulous, squamous, hyperpigmented lesions on light-exposed sites [220].

### 4.9. Antineoplastic Agents

Photosensitivity reactions have been reported for the following antineoplastic drugs:

(a) cytostatics: paclitaxel, fluorouracil, capecitabine, hydroxyurea, dacarbazine, vinblastine, 5-azacitidine;
(b) tyrosine kinase inhibitors: imatinib, vandetanib, vemurafenib, pazopanib;
(c) hormonal agents: flutamid, bicalutamide;
(d) monoclonal antibodies: mogamulizumab, rovalpituzumab.

Capecitabine may create a photosensitive lichenoid eruption [221]. Fluorouracil and paclitaxel are associated with subacute cutaneous lupus erythematosus [222,223]. Moreover, paclitaxel causes photodistributed rash, erythema, hyperpigmentation, and photoonycholysis [224,225]. Hydroxyurea induces phototoxicity reactions manifested by erythematous eruption that progresses to epidermal necrosis [226]. 5-azacitidine also causes phototoxicity. Clinical manifestations are pruritic, serpiginous, tender lesions [227].

The BRAF inhibitor, vemurafenib, induces phototoxicity under UVA radiation. Clinical manifestations are photodistributed erythema [228] and blistering sunburn [229]. The multitargeted tyrosine kinase inhibitors connected with photosensitivity are imatinib, pazopanib, and vandetanib. Imatinib causes erythematous edematous eruption limited to exposed skin and oral lichenoid reaction [230,231]. Pazopanib was shown to induce phototoxicity manifested by an exaggerated sunburn response [232]. Vandetanib use is connected with the following cutaneous lesions: exaggerated sunburn [233], photoinduced erythema multiforme [234], dark blue-grey pigmentation [235], and erythematous edematous [236].

The nonsteroidal antiandrogens, flutamide, and bicalutamide, have been shown to induce photosensitivity. The clinical manifestation of bicalutamide photosensitivity is edematous erythema involving light-exposed areas [237]. Flutamide causes a phototoxicity reaction manifested by exfoliative dermatitis followed by widespread vitiligo [238].

Mogamulizumab use is associated with photosensitive erythematous eruptions [239]. Rovalpituzumab induces phototoxic reactions with violaceous, erythematous, bullous patches [240].

#### 4.9.1. Fluorouracil

Fluorouracil has photosensitive potential under UVB irradiation. It has been reported that fluorouracil has a phototoxic effect on cultured fibroblasts and keratinocytes. Under UVB irradiation fluorouracil induces the formation of reactive species, in particular superoxide anion, and photooxidation of proteins. Moreover, UVB radiation determines fluorouracil-amino acid photoaddition that may result in a photoallergic reaction [241].

#### 4.9.2. Vandetanib

**Figure 10:**
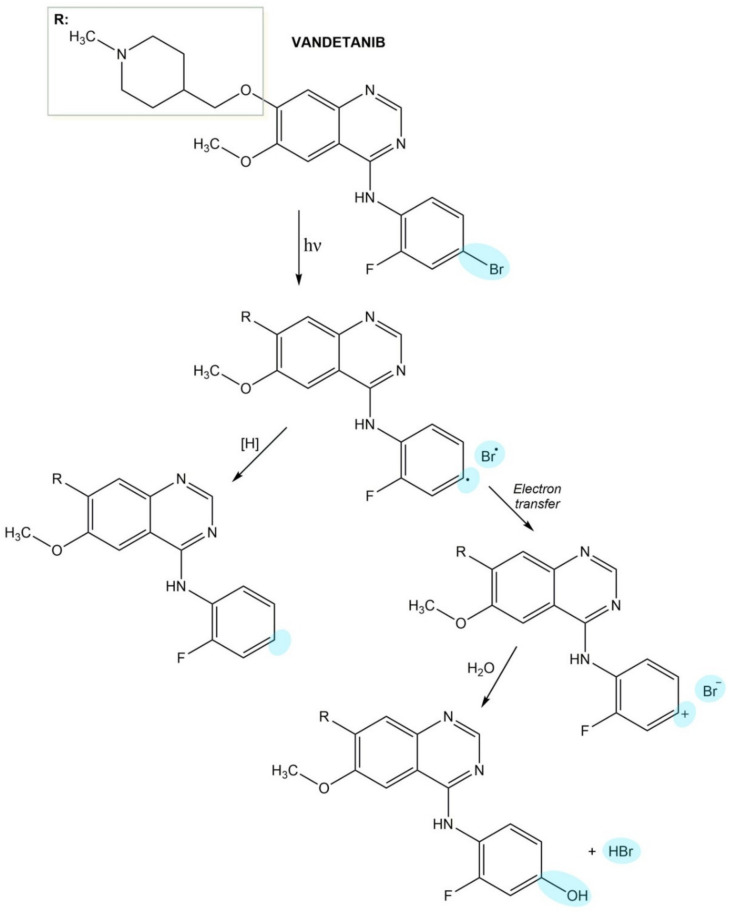
The photodegradation process of vandetanib.

UVA irradiation of vandetanib leads to C-Br bond homolysis and aryl radical formation. Aryl radical may accept the hydrogen atom and be converted to a stable photoproduct. In the alternative pathway, aryl radical may undergo electron transfer that results in the generation of very reactive aryl cation (Figure 10) [242].

### 4.10. Miscellaneous Drugs

Systemic retinoids, isotretinoin and etretinate, increase susceptibility to sunburn and induce photoleukomelanoderma [29].

Hormonal contraceptives are associated with photosensitivity manifested by erythematous skin lesions with vesicles [243].

The ulcer-healing agents inducing photosensitivity are ranitidine and proton pump inhibitors, such as esomeprazole. Ranitidine, under UVB irradiation, causes edematous erythematous eruptions with papules and vesicles [244]. The esomeprazole use is connected with photoallergy. Clinical manifestations are erythematous, scaly, pruritic patches on the sun-exposed areas that progress to ulcers [245].

Among drugs approved for the treatment of idiopathic pulmonary fibrosis, pirfenidone is shown to induce photosensitivity. Skin manifestations are the most common adverse effects of this drug. It is assumed that pathomechanism of pirfenidone photosensitivity involves photoallergy and phototoxicity. It induces erythematous, burning, pruritic lesions followed by hyperpigmentation [246,247].

## 5. Summary

**Figure 11:**
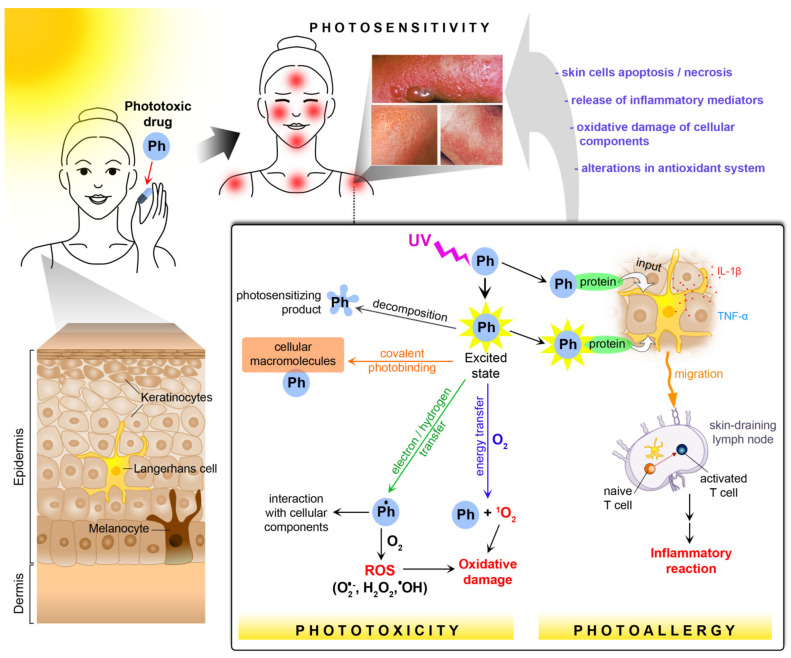
Mechanisms of drug-induced photosensitivity (Ph—phototoxic drug; ROS—reactive oxygen species).

Drug-induced photosensitivity is a serious medical problem. Many widely used drugs have been shown to induce phototoxic and/or photoallergic reactions. Moreover, the incidence of photosensitivity is constantly increasing. The final response to a photosensitive reaction is in fact a result of many different mechanisms and processes, which are summarized in Figure 11. The phototoxic potential of drugs depends on various factors, therefore, thorough assessment of photosafety is difficult. The following factors should be considered: the chemical properties of the drug, the influence of individual ranges of sunlight (in particular: UVA, UVB, visible light), the presence of melanin biopolymers, and the defense mechanisms of particular types of tested cells. The currently used methods of assessing the photosafety of drug seem to be preliminary. The complexity of the biological response to phototoxic reactions requires more detailed analyzes and the development of new models for testing drug-induced photosensitivity.

## Figures and Tables

**Table 1:** Artificial sources of ultraviolet radiation [20,21,22].

Source	Emitted Spectrum of Wavelengths
Germicidal lamps for sterilization and disinfection	UVC
Welding arcs	Full spectrum of ultraviolet (UVA, UVB, UVC)
High power metal halide and tungsten halogen lamps	Full spectrum of ultraviolet (UVA, UVB, UVC)
UV lasers and light emitting diodes (LEDs)	UVA/UVB/UVC
Fluorescent lamps	UVA + UVB
Sunlamps, sunbeds, and tanning beds	UVA or UVA + UVB
Phototherapy lamps used for medical and dental conditions	UVA or UVB
UV photocuring	UVA
UVA “blacklight” lamps	UVA
UVA lamps used for material inspection in industry, checking banknotes, and as insect traps	UVA

## Data Availability

Data sharing not applicable.

## References

[1] Nayak S., Acharjya B. (2008). Adverse cutaneous drug reaction. Ind. J. Dermatol..

[2] Carr D.F., Pirmohamed M. (2018). Biomarkers of adverse drug reactions. Exp. Biol. Med..

[3] Fiscus V., Hankinson A., Alweis R. (2014). Minocycline-induced hyperpigmentation. J. Community Hosp. Intern. Med. Perspect..

[4] Hoetzenecker W., Nägeli M., Mehra E.T., Jensen A.N., Saulite I., Schmid-Grendelmeier P., Guenova E., Cozzio A., French L.E. (2016). Adverse cutaneous drug eruptions: Current understanding. Seminars in Immunopathology.

[5] Sharma A.M., Uetrecht J. (2014). Bioactivation of drugs in the skin: Relationship to cutaneous adverse drug reactions. Drug Metab. Rev..

[6] Marzano A.V., Borghi A., Cugno M. (2016). Adverse drug reactions and organ damage: The skin. Eur. J. Intern. Med..

[7] Kiepurska N., Paluchowska E., Owczarek W., Szkultecka-Dębek M., Jahnz-Różyk K. (2017). The direct costs of drug-induced skin reactions. Ann. Agric. Environ. Med..

[8] Drucker A.M., Rosen C.F. (2011). Drug-induced photosensitivity: Culprit drugs, management and prevention. Drug Saf..

[9] Kutlubay Z., Sevim A., Engin B., Tüzün Y. (2014). Photodermatoses, including phototoxic and photoallergic reactions (internal and external). Clin. Dermatol..

[10] Hölzle E., Lehmann P., Neumann N. (2009). Phototoxic and photoallergic reactions. J. Dtsch. Dermatol. Ges..

[11] FDA (2015). S10 Photosafety Evaluation of Pharmaceuticals Guidance for Industry. https://www.fda.gov/media/85076/download.

[12] EMA (2012). S10 Guidance on Photosafety Evaluation of Pharmaceuticals. https://www.ema.europa.eu/en/documents/regulatory-procedural-guideline/ich-guideline-s10-guidance-photosafety-evaluation-pharmaceuticals-step-3_en.pdf.

[13] ICH (2013). Safety Guidelines S10 Photosafety Evaluation of Pharmaceuticals. https://database.ich.org/sites/default/files/S10_Guideline.pdf.

[14] Solano F. (2020). Photoprotection and skin pigmentation: Melanin-related molecules and some other new agents obtained from natural sources. Molecules.

[15] Shin D.W. (2020). Various biological effects of solar radiation on skin and their mechanisms: Implications for phototherapy. Anim. Cells Syst..

[16] Gould J.W., Mercurio M.G., Elmets C.A. (1995). Cutaneous photosensitivity diseases induced by exogenous agents. J. Am. Acad. Dermatol..

[17] Diffey B.L. (2002). Human exposure to solar ultraviolet radiation. J. Cosmet. Dermatol..

[18] Blumthaler M., Ambach W., Ellinger R. (1997). Increase in solar UV radiation with altitude. J. Photochem. Photobiol. B.

[19] Fioletov V., Kerr J.B., Fergusson A. (2010). The UV index: Definition, distribution and factors affecting it. Can. J. Public Health.

[20] IARC (2006). Exposure to Artificial UV Radiation and Skin Cancer. https://www.iarc.who.int/wp-content/uploads/2018/07/ArtificialUVRadSkinCancer.pdf.

[21] ICNIRP (2007). Protecting Workers from Ultraviolet Radiation. https://www.who.int/uv/publications/Protecting_Workers_UV_pub.pdf.

[22] Hietanen M. Occupational Exposure to Artificial Sources of UVR and Prevention. https://oshwiki.eu/wiki/Occupational_exposure_to_artificial_sources_of_UVR_and_prevention.

[23] Dubakiene R., Kupriene M. (2006). Scientific problems of photosensitivity. Medicina.

[24] González E., González S. (1997). Drug photosensitivity, idiopathic photodermatoses, and sunscreens. J. Am. Acad. Dermatol..

[25] Harber L.C., Baer R.L. (1972). Pathogenic mechanisms of drug-induced photosensitivity. J. Invest. Dermatol..

[26] Vassileva S.G., Mateev G., Parish L.C. (1998). Antimicrobial photosensitive reactions. Arch. Intern. Med..

[27] Quintero B., Miranda M.A. (2000). Mechanisms of photosensitization induced by drugs: A general survey. Ars Pharm..

[28] Ahmad I., Ahmed S., Anwar Z., Sheraz M.A., Sikorski M. (2016). Photostability and photostabilization of drugs and drug products. Int. J. Photoenergy.

[29] Monteiro A.F., Rato M., Martins C. (2016). Drug-induced photosensitivity: Photoallergic and phototoxic reactions. Clin. Dermatol..

[30] Stein K.R., Scheinfeld N.S. (2007). Drug-induced photoallergic and phototoxic reactions. Expert Opin. Drug Saf..

[31] Miranda M.A., Castell J.V., Gómez-Lechón M.J., Hernández D., Martínez L.A. (1995). Photobinding of drugs to cells as an indicator of potential photoallergy. Toxicol. In Vitro.

[32] Divkovic M., Pease C.K., Gerberick G.F., Basketter D.A. (2005). Hapten-protein binding: From theory to practical application in the in vitro prediction of skin sensitization. Contact Dermat..

[33] Boscá F., Cuquerella M.C., Marín M.L., Miranda M.A. (2001). Photochemistry of 2-hydroxy-4-trifluoromethylbenzoic acid, major metabolite of the photosensitizing platelet antiaggregant drug triflusal. Photochem. Photobiol..

[34] Caffieri S., Miolo G., Seraglia R., Dalzoppo D., Toma F.M., van Henegouwen G.M.B. (2007). Photoaddition of fluphenazine to nucleophiles in peptides and proteins. Possible cause of immune side effects. Chem. Res. Toxicol..

[35] Nuin E., Pérez-Sala D., Lhiaubet-Vallet V., Andreu I., Miranda M.A. (2016). Photosensitivity to triflusal: Formation of a photoadduct with ubiquitin demonstrated by photophysical and proteomic techniques. Front. Pharmacol..

[36] Cumberbatch M., Dearman R.J., Kimber I. (1999). Langerhans cell migration in mice requires intact type I interleukin 1 receptor (IL-1RI) function. Arch. Dermatol. Res..

[37] Enk A.H., Katz S.I. (1992). Early molecular events in the induction phase of contact sensitivity. Proc. Natl. Acad. Sci. USA.

[38] Sallusto F., Palermo B., Lenig D., Miettinen M., Matikainen S., Julkunen I., Forster R., Burgstahler R., Lipp M., Lanzavecchia A. (1999). Distinct patterns and kinetics of chemokine production regulate dendritic cell function. Eur. J. Immunol..

[39] Teunissen M.B.M. (1992). Dynamic nature and function of epidermal Langerhans cells in vivo and in vitro: A review, with emphasis on human Langerhans cells. Histochem. J..

[40] Toebak M.J., Gibbs S., Bruynzeel D.P., Scheper R.J., Rustemeyer T. (2009). Dendritic cells: Biology of the skin. Contact Dermatitis.

[41] Martins L.E.A.M., Reis V.M.S.D. (2011). Immunopathology of allergic contact dermatitis. An. Bras. Dermatol..

[42] Novak-Bilić G., Vučić M., Japundžić I., Meštrović-Štefekov J., Stanić-Duktaj S., Lugović-Mihić L. (2018). Irritant and allergic contact dermatitis—Skin lesion characteristics. Acta Clin. Croat..

[43] Kim K., Park H., Lim K.M. (2015). Phototoxicity: Its mechanism and animal alternative test methods. Toxicol. Res..

[44] Svensson C.K., Cowen E.W., Gaspari A.A. (2001). Cutaneous drug reactions. Pharmacol. Rev..

[45] Ibuki Y., Toyooka T. (2015). Evaluation of chemical phototoxicity, focusing on phosphorylated histone H2AX. J. Radiat. Res..

[46] Mang R., Stege H., Krutmann J., Johansen J., Frosch P., Lepoittevin J.P. (2011). Mechanisms of phototoxic and photoallergic reactions. Contact Dermatitis.

[47] Kulesza M., Dansonka-Mieszkowska A., Pieńkowska-Grela B. (2019). Napraw albo zgiń—Rola białka p53 w życiu komórki. Biul. Pol. Tow. Onkol. Nowotw..

[48] Trouba K.J., Hamadeh H.K., Amin R.P., Germolec D.R. (2002). Oxidative stress and its role in skin disease. Antioxid. Redox Signal..

[49] Marrot L., Meunier J.R. (2008). Skin DNA photodamage and its biological consequences. J. Am. Acad. Dermatol..

[50] Bickers D.R., Athar M. (2006). Oxidative stress in the pathogenesis of skin disease. J. Investig. Dermatol..

[51] Roux P.P., Blenis J. (2004). ERK and p38 MAPK-activated protein kinases: A family of protein kinases with diverse biological functions. Microbiol. Mol. Biol. Rev..

[52] Rutkowski R., Pancewicz S.A., Skrzydlewska E., Hermanowska-Szpakowicz T. (2005). Właściwości biologiczne czynnika transkrypcji jądrowej NF-κB. Alerg. Astma Immunol..

[53] Nicolaou A., Pilkington S.M., Rhodes L.E. (2011). Ultraviolet-radiation induced skin inflammation: Dissecting the role of bioactive lipids. Chem. Phys. Lipids.

[54] Rzepka Z., Buszman E., Beberok A., Wrześniok D. (2016). From tyrosine to melanin: Signaling pathways and factors regulating melanogenesis. Postepy Hig. Med. Dosw..

[55] D’Ischia M., Wakamatsu K., Cicoira F., di Mauro E., Garcia-Borron J.C., Commo S., Galván I., Ghanem G., Kenzo K., Meredith P. (2015). Melanins and melanogenesis: From pigment cells to human health and technological applications. Pigment Cell Melanoma Res..

[56] Park H.Y., Kosmadaki M., Yaar M., Gilchrest B.A. (2009). Cellular mechanisms regulating human melanogenesis. Cell. Mol. Life Sci..

[57] Brenner M., Hearing V.J. (2008). The protective role of melanin against UV damage in human skin. Photochem. Photobiol..

[58] Ito S., Wakamatsu K., Ozeki H. (2000). Chemical analysis of melanins and its application to the study of the regulation of melanogenesis. Pigment Cell Res..

[59] Slominski A., Tobin D.J., Shibahara S., Wortsman J. (2004). Melanin pigmentation in mammalian skin and its hormonal regulation. Physiol. Rev..

[60] ElObeid A.S., Kamal-Eldin A., Abdelhalim M.A.K., Haseeb A.M. (2017). Pharmacological properties of melanin and its function in health. Basic Clin. Pharmacol. Toxicol..

[61] Bustamante J., Bredeston L., Malanga G., Mordoh J. (1993). Role of melanin as a scavenger of active oxygen species. Pigment Cell Res..

[62] Meredith P., Sarna T. (2006). The physical and chemical properties of eumelanin. Pigment Cell Res..

[63] Napolitano A., Panzella L., Monfrecola G., d’Ischia M. (2014). Pheomelanin-induced oxidative stress: Bright and dark chemistry bridging red hair phenotype and melanoma. Pigment Cell Melanoma Res..

[64] Ortonne J.P. (2002). Photoprotective properties of skin melanin. Br. J. Dermatol..

[65] Simon J.D., Peles D., Wakamatsu K., Ito S. (2009). Current challenges in understanding melanogenesis bridging chemistry, biological control, morphology, and function. Pigment Cell Melanoma Res..

[66] Simon J.D., Hong L., Peles D.N. (2008). Insights into melanosomes and melanin from some interesting spatial and temporal properties. J. Phys. Chem. B.

[67] Maddodi N., Jayanthy A., Setaluri V. (2012). Shining light on skin pigmentation: The darker and the brighter side of effects of UV radiation. Photochem. Photobiol..

[68] Gupta A.K., Bharadwaj M., Mehrotra R. (2016). Skin cancer concerns in people of color: Risk factors and prevention. Asian Pac. J. Cancer Prev..

[69] Reinen J., van Sas P., van Huygevoort T., Rubio L., Scase K., Wenker M. (2018). Development of a phototoxicity testing strategy for accurate photosafety evaluation of pharmaceuticals based on the assessment of possible melanin-binding effects. Int. J. Toxicol..

[70] Beberok A., Buszman E., Wrześniok D., Otręba M., Trzcionka J. (2011). Interaction between ciprofloxacin and melanin: The effect on proliferation and melanization in melanocytes. Eur. J. Pharmacol..

[71] Buszman E., Wrześniok D., Trzcionka J., Miernik-Biela E., Stróż M. (2009). Interaction of ketoprofen and paracetamol with melanin in vitro. Ann. Univ. Mariae Curie Sklodowska Med..

[72] Rok J., Rzepka Z., Respondek M., Beberok A., Wrześniok D. (2019). Chlortetracycline and melanin biopolymer—The risk of accumulation and implications for phototoxicity: An in vitro study on normal human melanocytes. Chem. Biol. Interact..

[73] Rok J., Buszman E., Beberok A., Delijewski M., Otręba M., Wrześniok D. (2015). Modulation of melanogenesis and antioxidant status of melanocytes in response to phototoxic action of doxycycline. Photochem. Photobiol..

[74] Buszman E., Wrześniok D., Otręba M., Beberok A. (2012). The impact of ketoprofen on viability and melanization process in normal melanocytes HEMn-DP. Curr. Issues Pharm. Med. Sci..

[75] Rok J., Rzepka Z., Kowalska J., Banach K., Beberok A., Wrześniok D. (2021). Molecular and biochemical basis of minocycline-induced hyperpigmentation-the study on normal human melanocytes exposed to UVA and UVB radiation. Int. J. Mol. Sci..

[76] Rok J., Rzepka Z., Maszczyk M., Beberok A., Wrześniok D. (2021). Minocycline impact on redox homeostasis of normal human melanocytes HEMn-LP exposed to UVA radiation and hydrogen peroxide. Int. J. Mol. Sci..

[77] Onder G., Pellicciotti F., Gambassi G., Bernabei R. (2004). NSAID-related psychiatric adverse events: Who is at risk?. Drugs.

[78] Sánchez-Borges M., Capriles-Hulett A., Caballero-Fonseca F. (2005). Risk of skin reactions when using ibuprofen-based medicines. Expert Opin. Drug Saf..

[79] Bagheri H., Lhiaubet V., Montastruc J.L., Chouini-Lalanne N. (2000). Photosensitivity to ketoprofen: Mechanisms and pharmacoepidemiological data. Drug Saf..

[80] Canelas M.M., Cardoso J.C., Gonçalo M., Figueiredo A. (2010). Photoallergic contact dermatitis from benzydamine presenting mainly as lip dermatitis. Contact Derm..

[81] Cardoso J., Canelas M.M., Gonçalo M., Figueiredo A. (2009). Photopatch testing with an extended series of photoallergens: A 5-year study. Contact Derm..

[82] Varela P., Amorim I., Massa A., Sanches M., Silva E. (1998). Piroxicam-beta-cyclodextrin and photosensitivity reactions. Contact Derm..

[83] Serrano G., Bonillo J., Aliaga A., Cuadra J., Pujol C., Pelufo C., Cervera P., Miranda M.A. (1990). Piroxicam-induced photosensitivity and contact sensitivity to thiosalicylic acid. J. Am. Acad. Dermatol..

[84] Gonçalo M., Johansen J., Frosch P., Lepoittevin J.P. (2011). Phototoxic and photoallergic reactions. Contact Dermatitis.

[85] Akat P.B. (2013). Severe photosensitivity reaction induced by topical diclofenac. Indian J. Pharmacol..

[86] Karlsson I., Persson E., Ekebergh A., Mårtensson J., Börje A. (2014). Ketoprofen-induced formation of amino acid photoadducts: Possible explanation for photocontact allergy to ketoprofen. Chem. Res. Toxicol..

[87] Diaz R.L., Gardeazabal J., Manrique P., Ratón J.A., Urrutia I., Rodríguez-Sasiain J.M., Aguirre C. (2006). Greater allergenicity of topical ketoprofen in contact dermatitis confirmed by use. Contact Derm..

[88] Loh T.Y., Cohen P.R. (2016). Ketoprofen-induced photoallergic dermatitis. Ind. J. Med. Res..

[89] Monti S., Sortino S., de Guidib G., Marconi G. (1997). Photochemistry of 2-(3-benzoylphenyl)propionic acid (ketoprofen) Part 1 A picosecond and nanosecond time resolved study in aqueous solution. J. Chem. Soc. Faraday Trans..

[90] Martinez L.J., Scaiano J.C. (1997). Transient intermediates in the laser flash photolysis of ketoprofen in aqueous solutions: Unusual photochemistry for the benzophenone chromophore. J. Am. Chem. Soc..

[91] Nakazawa T., Shimo T., Chikamatsu N., Igarashi T., Nagata O., Yamamoto M. (2006). Study on the mechanism of photosensitive dermatitis caused by ketoprofen in the guinea pig. Arch. Toxikol..

[92] Shinoda M., Isozaki T., Suzuki T. (2014). Photoreaction of ketoprofen with tryptophan and tyrosine in phosphate buffer solution. Photochem. Photobiol..

[93] Atarashi K., Kabashima K., Akiyama K., Tokura Y. (2007). Stimulation of Langerhans cells with ketoprofen plus UVA in murine photocontact dermatitis to ketoprofen. J. Dermatol. Sci..

[94] Ray R.S., Mujtaba S.F., Dwivedi A., Yadav N., Verma A., Kushwaha H.N., Amar S.K., Goel S., Chopra D. (2013). Singlet oxygen mediated DNA damage induced phototoxicity by ketoprofen resulting in mitochondrial depolarization and lysosomal destabilization. Toxicology.

[95] Selvaag E., Thune P. (1997). Phototoxicity to sulphonamide-derived oral antidiabetics and diuretics: Investigations in hairless mice. Photodermatol. Photoimmunol. Photomed..

[96] Gómez-Bernal S., Alvarez-Pérez A., Rodríguez-Pazos L., Gutiérrez-González E., Rodríguez-Granados M., Toribio J. (2014). Photosensitivity due to thiazides. Actas Dermosifiliogr..

[97] Green J.J., Manders S.M. (2001). Pseudoporphyria. J. Am. Acad. Dermatol..

[98] Lowe G., Henderson C.L., Grau R.H., Hansen C.B., Sontheimer R.D. (2011). A systematic review of drug-induced subacute cutaneous lupus erythematosus. Br. J. Dermatol..

[99] Srivastava M., Rencic A., Diglio G., Santana H., Bonitz P., Watson R., Ha E., Anhalt G.J., Provost T.T., Nousari C.H. (2003). Drug-induced, Ro/SSA-positive cutaneous lupus erythematosus. Arch. Dermatol..

[100] Masuoka E., Bito T., Shimizu H., Nishigori C. (2011). Dysfunction of melanocytes in photoleukomelanoderma following photosensitivity caused by hydrochlorothiazide. Photodermatol. Photoimmunol. Photomed..

[101] Baran R., Juhlin L. (2002). Photoonycholysis. Photodermatol. Photoimmunol. Photomed..

[102] Johnston G.A., Coulson I.H. (2002). Thiazide-induced lichenoid photosensitivity. Clin. Exp. Dermatol..

[103] Breier F., Feldmann R., Pelzl M., Gschnait F. (1998). Pseudoporphyria cutanea tarda induced by furosemide in a patient undergoing peritoneal dialysis. Dermatology.

[104] Rutherford T., Sinclair R. (2007). Photo-onycholysis due to indapamide. Australas. J. Dermatol..

[105] Rodríguez Granados M.T., Abalde T., García Doval I., de la Torre C. (2004). Systemic photosensitivity to quinapril. J. Eur. Acad. Dermatol. Venereol..

[106] Kanwar A.J., Dhar S., Ghosh S. (1993). Photosensitive lichenoid eruption due to enalapril. Dermatology.

[107] Frye C.B., Pettigrew T.J. (1998). Angioedema and photosensitive rash induced by valsartan. Pharmacotherapy.

[108] Bakkour W., Haylett A.K., Gibbs N.K., Chalmers R.J., Rhodes L.E. (2013). Photodistributed telangiectasia induced by calcium channel blockers: Case report and review of the literature. Photodermatol. Photoimmunol. Photomed..

[109] Basarab T., Yu R., Russell Jones R. (1997). Calcium antagonist-induced photo-exposed telangiectasia. Br. J. Dermatol..

[110] Ramírez A., Pérez-Pérez L., Fernández-Redondo V., Toribio J. (2007). Photoallergic dermatitis induced by diltiazem. Contact Derm..

[111] Scherschun L., Lee M.W., Lim H.W. (2001). Diltiazem-associated photodistributed hyperpigmentation: A review of 4 cases. Arch. Dermatol..

[112] Boyer M., Katta R., Markus R. (2003). Diltiazem-induced photodistributed hyperpigmentation. Dermatol. Online J..

[113] Miyauchi H., Horiki S., Horio T. (1994). Clinical and experimental photosensitivity reaction to tilisolol hydrochloride. Photodermatol. Photoimmunol. Photomed..

[114] Vassallo P., Trohman R.G. (2007). Prescribing amiodarone: An evidence-based review of clinical indications. JAMA.

[115] Jaworski K., Walecka I., Rudnicka L., Gnatowski M., Kosior D.A. (2014). Cutaneous adverse reactions of amiodarone. Med. Sci. Monit..

[116] Yones S.S., O’Donoghue N.B., Palmer R.A., Menagé H.D.P., Hawk J.L.M. (2005). Persistent severe amiodarone-induced photosensitivity. Clin. Exp. Dermatol..

[117] Harris L., McKenna W.J., Rowland E., Holt D.W., Storey G.C.A., Krikler D.M. (1983). Side effects of long-term amiodarone therapy. Circulation.

[118] Joshi K.M., Gill M.K. (2016). Amiodarone: A potential risk factor for retinal phototoxicity. Am. J. Ophthalmol. Case Rep..

[119] Martínez Leboráns L., Cubells Sánchez L., Zaragoza Ninet V., Pérez Ferriols A. (2016). Atypical photosensitivity associated with triflusal. Contact Derm..

[120] Dogra S., Kanwar A.J. (2003). Clopidogrel bisulphate-induced photosensitive lichenoid eruption: First report. Br. J. Dermatol..

[121] Mota A.V., Vasconcelos C., Correia T.M., Barros M.A., Mesquita-Guimarães J. (1998). Rilmenidine-induced photosensitivity reaction. Photodermatol. Photoimmunol. Photomed..

[122] Vaillant L., Le Marchand D., Grognard C., Hocine R., Lorette G. (1988). Photosensitivity to methyldopa. Arch. Dermatol..

[123] Matsuo I., Fujita H., Hayakawa K., Ohkido M. (1986). Lipid peroxidative potency of photosensitized thiazide diuretics. J. Invest. Dermatol..

[124] Kunisada M., Masaki T., Ono R., Morinaga H., Nakano E., Yogianti F., Okunishi K., Sugiyama H., Nishigori C. (2013). Hydrochlorothiazide enhances UVA-induced DNA damage. Photochem. Photobiol..

[125] Han K.D., Bark K.M., Heo E.P., Lee J.K., Kang J.S., Kim T.H. (2000). Increased phototoxicity of hydrochlorothiazide by photodegradation. Photodermatol. Photoimmunol. Photomed..

[126] Onoue S., Igarashi N., Yamada S., Tsuda Y. (2008). High-throughput reactive oxygen species (ROS) assay: An enabling technology for screening the phototoxic potential of pharmaceutical substances. J. Pharm. Biomed. Anal..

[127] Al-Ajmi H.S., Dawe R.S., Renwick A.G., Macklin B.S., Ferguson J., Gibbs N.K. (2000). The effect of whole-body sunbed ultraviolet A exposure on the pharmacokinetics of the photolabile drug nifedipine. Photodermatol. Photoimmunol. Photomed..

[128] De Vries H., van Henegouwen G.M.B. (1998). Photoreactivity of nifedipine in vitro and in vivo. J. Photochem. Photobiol. B.

[129] Boscá F., Miranda M.A. (1998). Photosensitizing drugs containing the benzophenone chromophore. J. Photochem. Photobiol. B.

[130] Marguery M.C., Chouini-Lalanne N., Drugeon C., Gadroy A., Bayle P., Journe F., Bazex J., D’Incan M. (2006). UV-B phototoxic effects induced by atorvastatin. Arch. Dermatol..

[131] Granados M.T., de la Torre C., Cruces M.J., Piñeiro G. (1998). Chronic actinic dermatitis due to simvastatin. Contact Derm..

[132] Rodríguez-Pazos L., Sánchez-Aguilar D., Rodríguez-Granados M., Pereiro-Ferreirós M., Toribio J. (2010). Erythema multiforme photoinduced by statins. Photodermatol. Photoimmunol. Photomed..

[133] Holme S.A., Pearse A.D., Anstey A.V. (2002). Chronic actinic dermatitis secondary to simvastatin. Photodermatol. Photoimmunol. Photomed..

[134] Mohammed F., Wally L.L., Karaban J.E., Reddy V.B., Lertratanakul Y. (2017). Fenofibrate-induced lichenoid drug eruption: A rare culprit. Case Rep. Dermatol..

[135] Tsai K.C., Yang J.H., Hung S.J. (2017). Fenofibrate-induced photosensitivity—A case series and literature review. Photodermatol. Photoimmunol. Photomed..

[136] Marguery M.C., Chouini-Lalanne N., Ader J.C., Paillous N. (1998). Comparison of the DNA damage photoinduced by fenofibrate and ketoprofen, two phototoxic drugs of parent structure. Photochem. Photobiol..

[137] Vayá I., Andreu I., Monje V.T., Jiménez M.C., Miranda M.A. (2016). Mechanistic studies on the photoallergy mediated by fenofibric acid: Photoreactivity with serum albumins. Chem. Res. Toxicol..

[138] Terencio M.C., Guillén I., Gómez-Lechón M.J., Miranda M.A., Castell J.V. (1998). Release of inflammatory mediators (PGE2, IL-6) by fenofibric acid-photosensitized human keratinocytes and fibroblasts. Photochem. Photobiol..

[139] Diemer S., Eberlein-König B., Przybilla B. (1996). Evaluation of the phototoxic properties of some hypolipidemics in vitro: Fenofibrate exhibits a prominent phototoxic potential in the UVA and UVB region. J. Dermatol. Sci..

[140] Lhiaubet V., Paillous N., Chouini-Lalanne N. (2001). Comparison of DNA damage photoinduced by ketoprofen, fenofibric acid and benzophenone via electron and energy transfer. Photochem. Photobiol..

[141] Miolo G., Levorato L., Gallocchio F., Caffieri S., Bastianon C., Zanoni R., Reddi E. (2006). In vitro phototoxicity of phenothiazines: Involvement of stable UVA photolysis products formed in aqueous medium. Chem. Res. Toxicol..

[142] Llambrich A., Lecha M. (2004). Photoinduced lichenoid reaction by thioridazine. Photodermatol. Photoimmunol. Photomed..

[143] Barbaud A., Collet E., Martin S., Granel F., Trechot P., Lambert D., Schmutz J.L. (2001). Contact sensitization to chlorproethazine can induce persistent light reaction and cross-photoreactions to other phenothiazines. Contact Derm..

[144] Giomi B., Difonzo E.M., Lotti L., Massi D., Francalanci S. (2011). Allergic and photoallergic conditions from unusual chlorpromazine exposure: Report of three cases. Int. J. Dermatol..

[145] Gacías L., Linares T., Escudero E., Soto-Mera M.T., Abalde M.T. (2013). Perphenazine as a cause of mother-to-daughter contact dermatitis and photocontact dermatitis. J. Investig. Allergol. Clin. Immunol..

[146] Romita P., Foti C., Stingeni L. (2017). Photoallergy to promazine hydrochloride. Contact Derm..

[147] Fernandes I.C., Vilaça S., Lobo I., Sanches M., Costa V., Selores M. (2013). Photoallergic reaction to cyamemazine. Dermatol. Online J..

[148] Bourrain J.L., Paillet C., Woodward C., Beani J.C., Amblard P. (1997). Diagnosis of photosensitivity to flupenthixol by photoprick testing. Photodermatol. Photoimmunol. Photomed..

[149] Gregoriou S., Karagiorga T., Stratigos A., Volonakis K., Kontochristopoulos G., Rigopoulos D. (2008). Photo-onycholysis caused by olanzapine and aripiprazole. J. Clin. Psychopharmacol..

[150] Ming M.E., Bhawan J., Stefanato C.M., McCalmont T.H., Cohen L.M. (1999). Imipramine-induced hyperpigmentation: Four cases and a review of the literature. J. Am. Acad. Dermatol..

[151] Thédenat B., Loche F., Albes B., Marguery M.C., Bazex J. (2001). Acute generalized exanthematous pustulosis with photodistribution pattern induced by sertraline. Dermatology.

[152] Doffoel-Hantz V., Boulitrop-Morvan C., Sparsa A., Bonnetblanc J.M., Dalac S., Bédane C. (2009). Photosensitivity associated with selective serotonin reuptake inhibitors. Clin. Exp. Dermatol..

[153] Ram-Wolf C., Mahé E., Saiag P. (2008). Escitalopram photo-induced erythroderma. J. Eur. Acad. Dermatol. Venereol..

[154] Inalöz H.S., Kirtak N., Herken H., Ozgöztaşi O., Aynacioğlu A.S. (2001). Citalopram-induced photopigmentation. J. Dermatol..

[155] Pazzagli L., Banfi R., Borselli G., Semmola M.V. (1998). Photosensitivity reaction to fluoxetine and alprazolam. Pharm. World Sci..

[156] Röhrs S., Geiser F., Conrad R. (2012). Citalopram-induced subacute cutaneous lupus erythematosus—First case and review concerning photosensitivity in selective serotonin reuptake inhibitors. Gen. Hosp. Psychiatry.

[157] Vaccaro M., Borgia F., Barbuzza O., Guarneri B. (2007). Photodistributed eruptive telangiectasia: An uncommon adverse drug reaction to venlafaxine. Br. J. Dermatol..

[158] Watanabe Y., Kawada A., Ohnishi Y., Tajima S., Ishibashi A. (1999). Photosensitivity due to alprazolam with positive oral photochallenge test after 17 days administration. J. Am. Acad. Dermatol..

[159] Chignell C.F., Motten A.G., Buettner G.R. (1985). Photoinduced free radicals from chlorpromazine and related phenothiazines: Relationship to phenothiazine-induced photosensitization. Environ. Health Perspect..

[160] Piñero-Santiago L.E., García C., Lhiaubet-Vallet V., Trzcionka J., Oyola R., Torres K., Leguillú J., Miranda M.A. (2013). Photooxidation mechanism of levomepromazine in different solvents. Photochem. Photobiol..

[161] Rodrigues T., dos Santos C.G., Riposati A., Barbosa L.R., di Mascio P., Itri R., Baptista M.S., Nascimento O.R., Nantes I.L. (2006). Photochemically generated stable cation radical of phenothiazine aggregates in mildly acid buffered solutions. J. Phys. Chem. B.

[162] Bacellar I.O., Pavani C., Sales E.M., Itri R., Wainwright M., Baptista M.S. (2014). Membrane damage efficiency of phenothiazinium photosensitizers. Photochem. Photobiol..

[163] Elisei F., Latterini L., Aloisi G.G., Mazzucato U., Viola G., Miolo G., Vedaldi D., Dall’Acqua F. (2002). Excited-state properties and in vitro phototoxicity studies of three phenothiazine derivatives. Photochem. Photobiol..

[164] Onoue S., Kato M., Inoue R., Seto Y., Yamada S. (2014). Photosafety screening of phenothiazine derivatives with combined use of photochemical and cassette-dosing pharmacokinetic data. Toxicol. Sci..

[165] Viola G., Latterini L., Vedaldi D., Aloisi G.G., Dall’Acqua F., Gabellini N., Elisei F., Barbafina A. (2003). Photosensitization of DNA strand breaks by three phenothiazine derivatives. Chem. Res. Toxicol..

[166] Drago F., Gasparini G., Marenco S., Picciotto A., Parodi A. (2016). Porphyrin elevation in a patient on treatment with simeprevir: Could it be a possible explanation for simeprevir-associated photosensitivity?. Am. J. Gastroenterol..

[167] Verma R., Vasudevan B., Shankar S., Pragasam V., Suwal B., Venugopal R. (2012). First reported case of tenofovir-induced photoallergic reaction. Ind. J. Pharmacol..

[168] Furue M. (2004). Photosensitive drug eruption induced by efavirenz in a patient with HIV infection. Intern. Med..

[169] Isaacs T., Ngwanya M.R., Dlamini S., Lehloenya R.J. (2013). Annular erythema and photosensitivity as manifestations of efavirenz-induced cutaneous reactions: A review of five consecutive cases. J. Antimicrob. Chemother..

[170] Treudler R., Husak R., Raisova M., Orfanos C.E., Tebbe B. (2001). Efavirenz-induced photoallergic dermatitis in HIV. AIDS.

[171] Yoshimoto E., Konishi M., Takahashi K., Murakawa K., Maeda K., Mikasa K., Yamashina Y. (2004). The first case of efavirenz-induced photosensitivity in a Japanese patient with HIV infection. Intern. Med..

[172] Alvarez-Fernández J.G., Castaño-Suárez E., Cornejo-Navarro P., de la Fuente E.G., de Frutos F.J.O., Iglesias-Diez L. (2000). Photosensitivity induced by oral itraconazole. J. Eur. Acad. Dermatol. Venereol..

[173] Lazzarini R., Hafner M.F.S., Miguel B.A.F., Kawakami N.T., Nakagome B.H.Y. (2018). Allergic contact dermatitis caused by topical ketoconazole: A relevant issue? Review of ketoconazole-positive patch tests. Contact Derm..

[174] Barbosa N.S., Wetter D.A. (2014). Bullous phototoxicity from voriconazole. J. Emerg. Med..

[175] Tolland J.P., McKeown P.P., Corbett J.R. (2007). Voriconazole-induced pseudoporphyria. Photodermatol. Photoimmunol. Photomed..

[176] Rubenstein M., Levy M.L., Metry D. (2004). Voriconazole-induced retinoid-like photosensitivity in children. Pediatr. Dermatol..

[177] Racette A.J., Roenigk H.H., Hansen R., Mendelson D., Park A. (2005). Photoaging and phototoxicity from long-term voriconazole treatment in a 15-year-old girl. J. Am. Acad. Dermatol..

[178] Miller D.D., Cowen E.W., Nguyen J.C., McCalmont T.H., Fox L.P. (2010). Melanoma associated with long-term voriconazole therapy: A new manifestation of chronic photosensitivity. Arch. Dermatol..

[179] Cowen E.W., Nguyen J.C., Miller D.D., McShane D., Arron S.T., Prose N.S., Turner M.L., Fox L.P. (2010). Chronic phototoxicity and aggressive squamous cell carcinoma of the skin in children and adults during treatment with voriconazole. J. Am. Acad. Dermatol..

[180] McCarthy K.L., Playford E.G., Looke D.F., Whitby M. (2007). Severe photosensitivity causing multifocal squamous cell carcinomas secondary to prolonged voriconazole therapy. Clin. Infect. Dis..

[181] Williams K., Mansh M., Chin-Hong P., Singer J., Arron S.T. (2014). Voriconazole-associated cutaneous malignancy: A literature review on photocarcinogenesis in organ transplant recipients. Clin. Infect. Dis..

[182] Ramachandran S.M., Leventhal J.S., Franco L.G., Mir A., Walters R.F., Franks A.G. (2017). Topical drug-induced subacute cutaneous lupus erythematosus isolated to the hands. Lupus Sci. Med..

[183] Morlière P., Silva A., Seixas R., Boscá F., Mazière J.C., Ferreira J., Santus R., Filipe P. (2018). Photosensitisation by voriconazole-N-oxide results from a sequence of solvent and pH-dependent photochemical and thermal reactions. J. Photochem. Photobiol. B.

[184] Ona K., Oh D.H. (2015). Voriconazole N-oxide and its ultraviolet B photoproduct sensitize keratinocytes to ultraviolet A. Br. J. Dermatol..

[185] Moore D.E. (2002). Drug-induced cutaneous photosensitivity: Incidence, mechanism, prevention and management. Drug Saf..

[186] Goetze S., Hiernickel C., Elsner P. (2017). Phototoxicity of doxycycline: A systematic review on clinical manifestations, frequency, cofactors, and prevention. Skin Pharmacol. Physiol..

[187] Yong C.K., Prendiville J., Peacock D.L., Wong L.T., Davidson A.G. (2000). An unusual presentation of doxycycline-induced photosensitivity. Pediatrics.

[188] Yap L.M., Foley P.A., Crouch R.B., Baker C.S. (2000). Drug-induced solar urticaria due to tetracycline. Australas. J. Dermatol..

[189] Owens R.C., Ambrose P.G. (2005). Antimicrobial safety: Focus on fluoroquinolones. Clin. Infect. Dis..

[190] Borgia F., Vaccaro M., Guarneri F., Cannavò S.P. (2000). Photodistributed telangiectasia following use of cefotaxime. Br. J. Dermatol..

[191] Lee A.Y., Jung S.Y. (1998). Two patients with isoniazid-induced photosensitive lichenoid eruptions confirmed by photopatch test. Photodermatol. Photoimmunol. Photomed..

[192] Katiyar S.K., Bihari S., Prakash S. (2010). Pyrazinamide-induced phototoxicity: A case report and review of literature. Ind. J. Dermatol..

[193] Kar B.R. (2008). Dapsone-induced photosensitivity: A rare clinical presentation. Photodermatol. Photoimmunol. Photomed..

[194] De D., Dogra S., Kaur I. (2007). Dapsone induced acute photosensitivity dermatitis; A case report and review of literature. Lepr. Rev..

[195] Cuquerella M.C., Miranda M.A., Bosca F. (2006). Generation of detectable singlet aryl cations by photodehalogenation of fluoroquinolones. J. Phys. Chem. B.

[196] Fasani E., Mella M., Caccia D., Tassi S., Fagnoni M., Albini A. (1997). The photochemistry of lomefloxacin. An aromatic carbene as the key intermediate in photodecomposition. Chem. Commun..

[197] Thompson A.M. (2007). Ocular toxicity of fluoroquinolones. Clin. Exp. Ophthalmol..

[198] Martínez L.J., Sik R.H., Chignell C.F. (1998). Fluoroquinolone antimicrobials: Singlet oxygen, superoxide and phototoxicity. Photochem. Photobiol..

[199] Shimoda K. (1998). Mechanisms of quinolone phototoxicity. Toxicol. Lett..

[200] De Guidi G., Bracchitta G., Catalfo A. (2011). Photosensitization reactions of fluoroquinolones and their biological consequences. Photochem. Photobiol..

[201] Kowalska J., Banach K., Rok J., Beberok A., Rzepka Z., Wrześniok D. (2020). Molecular and biochemical basis of fluoroquinolones-induced phototoxicity-the study of antioxidant system in human melanocytes exposed to UV-A radiation. Int. J. Mol. Sci..

[202] Viola G., Facciolo L., Canton M., Vedaldi D., Dall’Acqua F., Aloisi G.G., Amelia M., Barbafina A., Elisei F., Latterini L. (2004). Photophysical and phototoxic properties of the antibacterial fluoroquinolones levofloxacin and moxifloxacin. Chem. Biodivers.

[203] Soldevila S., Cuquerella M.C., Bosca F. (2014). Understanding of the photoallergic properties of fluoroquinolones: Photoreactivity of lomefloxacin with amino acids and albumin. Chem. Res. Toxicol..

[204] Nelson M.L. (1998). Chemical and biological dynamics of tetracyclines. Adv. Dent. Res..

[205] Chen Y., Hu C., Qu J., Yang M. (2008). Photodegradation of tetracycline and formation of reactive oxygen species in aqueous tetracycline solution under simulated sunlight irradiation. J. Photochem. Photobiol. A.

[206] Drexel R.E., Olack G., Jones C., Chmurny G.N., Santini R., Morrison H. (1990). Lumitetracycline: A novel new tetracycline photoproduct. J. Org. Chem..

[207] Shea C.R., Olack G.A., Morrison H., Chen N., Hasan T. (1993). Phototoxicity of lumidoxycycline. J. Invest. Dermatol..

[208] Chen Y., Li H., Wang Z., Tao T., Hu C. (2011). Photoproducts of tetracycline and oxytetracycline involving self-sensitized oxidation in aqueous solutions: Effects of Ca^2+^ and Mg^2+^. J. Environ. Sci..

[209] Chen Y., Li H., Wang Z., Tao T., Wei D., Hu C. (2012). Photolysis of chlortetracycline in aqueous solution: Kinetics, toxicity and products. J. Environ. Sci..

[210] Wammer K.H., Slattery M.T., Stemig A.M., Ditty J.L. (2011). Tetracycline photolysis in natural waters: Loss of antibacterial activity. Chemosphere.

[211] Jiao S., Zheng S., Yin D., Wang L., Chen L. (2008). Aqueous photolysis of tetracycline and toxicity of photolytic products to luminescent bacteria. Chemosphere.

[212] Epstein J.H. (1999). Phototoxicity and photoallergy. Semin. Cutan. Med. Surg..

[213] Aronson J.K. (2006). Meyler’s side effects of drugs. The International Encyclopedia of Adverse Drug Reactions and Interactions.

[214] Nicolaidou E., Katsambas A.D. (2014). Pigmentation disorders: Hyperpigmentation and hypopigmentation. Clin. Dermatol..

[215] Trzcionka J., Buszman E. (2003). Oddziaływanie antybiotyków tetracyklinowych z melaniną w aspekcie ich fototoksycznego działania na skórę. Ann. Acad. Med. Siles..

[216] Rok J., Buszman E., Delijewski M., Otręba M., Beberok A., Wrześniok D. (2015). Effect of tetracycline and UV radiation on melanization and antioxidant status of melanocytes. J. Photochem. Photobiol. B.

[217] Rok J., Wrześniok D., Beberok A., Otręba M., Delijewski M., Buszman E. (2018). Phototoxic effect of oxytetracycline on normal human melanocytes. Toxicol. In Vitro.

[218] Agudo-Mena J.L., Romero-Pérez D., Encabo-Durán B., Álvarez-Chinchilla P.J., Silvestre-Salvador J.F. (2017). Photoallergic contact dermatitis caused by quinidine sulfate in a caregiver. Contact Derm..

[219] Nacher M., McGready R., Lermoo C., Wichiponpiboon J., Nosten F. (2005). Photoallergy to quinine. Trop. Doct..

[220] Lisi P., Assalve D., Hansel K. (2004). Phototoxic and photoallergic dermatitis caused by hydroxychloroquine. Contact Derm..

[221] Walker G., Lane N., Parekh P. (2014). Photosensitive lichenoid drug eruption to capecitabine. J. Am. Acad. Dermatol..

[222] Almagro B.M., Steyls M.C., Navarro N.L., Domínguez E.G., Acosta E.H., Pérez M.A.G., Ceballos E.H. (2011). Occurrence of subacute cutaneous lupus erythematosus after treatment with systemic fluorouracil. J. Clin. Oncol..

[223] Weger W., Kränke B., Gerger A., Salmhofer W., Aberer E. (2008). Occurrence of subacute cutaneous lupus erythematosus after treatment with fluorouracil and capecitabine. J. Am. Acad. Dermatol..

[224] Beutler B.D., Cohen P.R. (2015). Nab-paclitaxel-associated photosensitivity: Report in a woman with non-small cell lung cancer and review of taxane-related photodermatoses. Dermatol. Pract. Concept..

[225] Hussain S., Anderson D.N., Salvatti M.E., Adamson B., McManus M., Braverman A.S. (2000). Onycholysis as a complication of systemic chemotherapy: Report of five cases associated with prolonged weekly paclitaxel therapy and review of the literature. Cancer.

[226] Yanamandra U., Sahu K.K., Malhotra P., Varma S. (2014). Photodermatosis secondary to hydroxyurea. BMJ Case Rep..

[227] Keating M., Dasanu C.A. (2017). Severe phototoxic reaction secondary to subcutaneous 5-azacitidine. J. Oncol. Pharm. Pract..

[228] Gabeff R., Dutartre H., Khammari A., Boisrobert A., Nguyen J.M., Quereux G., Brocard A., Saint-Jean M., Peuvrel L., Dreno B. (2015). Phototoxicity of B-RAF inhibitors: Exclusively due to UVA radiation and rapidly regressive. Eur. J. Dermatol..

[229] Jew O.S., Provini L.E., Treat J.R. (2019). Severe vemurafenib-induced photosensitivity in a 6-year-old boy. Pediatr. Dermatol..

[230] Brazzelli V., Muzio F., Manna G., Moggio E., Vassallo C., Orlandi E., Fiandrino G., Lucioni M., Borroni G. (2012). Photoinduced dermatitis and oral lichenoid reaction in a chronic myeloid leukemia patient treated with imatinib mesylate. Photodermatol. Photoimmunol. Photomed..

[231] Rousselot P., Larghero J., Raffoux E., Calvo F., Tulliez M., Giraudier S., Rybojad M. (2003). Photosensitization in chronic myelogenous leukaemia patients treated with imatinib mesylate. Br. J. Haematol..

[232] Udompanich S., Chanprapaph K., Rajatanavin N. (2018). Phototoxic reaction induced by pazopanib. Case Rep. Dermatol..

[233] Giacchero D., Ramacciotti C., Arnault J.P., Brassard M., Baudin E., Maksimovic L., Mateus C., Tomasic G., Wechsler J., Schlumberger M. (2012). A new spectrum of skin toxic effects associated with the multikinase inhibitor vandetanib. Arch. Derm..

[234] Caro-Gutiérrez D., Floristán Muruzábal M.U., de la Fuente E.G., Franco A.P., López Estebaranz J.L. (2014). Photo-induced erythema multiforme associated with vandetanib administration. J. Am. Acad. Dermatol..

[235] Kong H.H., Fine H.A., Stern J.B., Turner M.L.C. (2009). Cutaneous pigmentation after photosensitivity induced by vandetanib therapy. Arch. Derm..

[236] Fava P., Quaglino P., Fierro M.T., Novelli M., Bernengo M.G. (2010). Therapeutic hotline. A rare vandetanib-induced photo-allergic drug eruption. Dermatol. Ther..

[237] Lee K., Oda Y., Sakaguchi M., Yamamoto A., Nishigori C. (2016). Drug-induced photosensitivity to bicalutamide—Case report and review of the literature. Photodermatol. Photoimmunol. Photomed..

[238] Rafael J.P., Manuel G.G., Antonio V., Carlos M.J. (2004). Widespread vitiligo after erythroderma caused by photosensitivity to flutamide. Contact Derm..

[239] Masuda Y., Tatsuno K., Kitano S., Miyazawa H., Ishibe J., Aoshima M., Shimauchi T., Fujiyama T., Ito T., Tokura Y. (2018). Mogamulizumab-induced photosensitivity in patients with mycosis fungoides and other T-cell neoplasms. J. Eur. Acad. Dermatol. Venereol..

[240] Hou J.L., Bridges A.G. (2018). Phototoxic drug reaction with the novel agent rovalpituzumab tesirine. Int. J. Dermatol..

[241] Miolo G., Marzano C., Gandin V., Palozzo A.C., Dalzoppo D., Salvador A., Caffieri S. (2011). Photoreactivity of 5-fluorouracil under UVB light: Photolysis and cytotoxicity studies. Chem Res Toxicol..

[242] Dall’acqua S., Vedaldi D., Salvador A. (2013). Isolation and structure elucidation of the main UV-A photoproducts of vandetanib. J. Pharm. Biomed. Anal..

[243] Gómez-Bernal S., Loureiro M., Rodríguez-Granados M.T., Toribio J. (2010). Systemic photosensitivity due to a contraceptive patch. Photodermatol. Photoimmunol. Photomed..

[244] Kondo S., Kagaya M., Yamada Y., Matsusaka H., Jimbow K. (2000). UVB photosensitivity due to ranitidine. Dermatology.

[245] Shukla A., Mahapatra A., Gogtay N., Khopkar U. (2010). Esomeprazole-induced photoallergic dermatitis. J. Postgrad. Med..

[246] Droitcourt C., Adamski H., Polat A., Polard E., Kerjouan M., Arnouat B., Garrec M.L., Oger E., Dupuy A., Jouneau S. (2018). Pirfenidone photosensitization in patients with idiopathic pulmonary fibrosis: A case series. Br. J. Dermatol..

[247] Park M.-Y., Shim W.-H., Kim J.-M., Kim G.-W., Kim H.-S., Ko H.-C., Kim M.-B., Kim B.-S. (2017). Pirfenidone-induced photo-allergic reaction in a patient with idiopathic pulmonary fibrosis. Photodermatol. Photoimmunol. Photomed..

